# Unraveling carbonate fault dynamics, from friction to decarbonation, through the 1959 Mw 7.2 earthquake in Montana

**DOI:** 10.1038/s41598-025-89071-4

**Published:** 2025-03-13

**Authors:** Nina Zamani, Michael A. Murphy, Eric C. Ferré, Fabrice Barou

**Affiliations:** 1 Department of Earth & Atmospheric Sciences, University of Houston, Houston, TX 77204 USA; 2 Department of Geological Sciences, New Mexico State University, Las Cruces, NM 88003 USA; 3 Géosciences Montpellier UMR CNRS 5243, Université de Montpellier, 34095 Montpelier, France

**Keywords:** Seismic rupture, Carbonate rocks, Fault friction, Coseismic heating, Iron oxides, Decarbonation, West Yellowstone, Solid Earth sciences, Tectonics

## Abstract

Seismic rupture in carbonate rocks influences fault friction behavior through thermal evolution and mineral reactions. Focusing on the 1959 Mw 7.2 Hebgen Lake event in western Yellowstone, Montana, the largest earthquake on a normal fault in the United States, we analyze fault rock microstructures and mineralogical changes to constrain frictional heating on the fault plane. We combine thermal maturity of organic matter, magnetic fabric, and thermomagnetic methods with scanning electron microscopy to unravel variations in peak frictional temperature along the fault slip surface. The mineral changes caused by coseismic heating (e.g., nanocalcite formation or goethite to hematite reaction) occur in patches along the fault mirror, hence reflecting considerable differences in frictional heat. While coseismic thermal heterogeneities have been reported in other rock types, this is the first time they are documented and quantified specifically in carbonates. Furthermore, these results provide new mineralogical criteria to quantify coseismic frictional heat in natural faults at temperatures lower than that of decarbonation and highlight the need to consider coseismic friction processes at a scale larger than most deformation experiments. For example, we document the critical role played by fault plane attitude (dip) at the scale of a few tens of centimeters in production of frictional heat. Our results emphasize that while coseismic decarbonation dynamically weakens carbonate-hosted faults, it may generally not occur along an entire fault plane.

## Introduction

Earthquake energy is dissipated mainly along the fault plane in the form of frictional heat and rock fracturing and, to a lesser extent, as radiated seismic waves^1,2^ resulting in destructive effects on population centers^3^. Multiple meso- and microstructures indicate slip at seismic velocities^4^. Frictional heating along faults is documented through various indicators such as clay mineral reactions^5,6^, fission tracks^7^, or mineral dehydration^8^. In some cases, coseismic frictional heat is so intense that it results in frictional melting, i.e., the formation of pseudotachylytes^9^. In carbonates, instead of melting, frictional heating leads to calcite (or dolomite) breakdown into lime (or lime and periclase) and carbon dioxide. Due to the transient nature of frictional heating and lack of simple methods to measure the heat budget of seismic deformation, the heat budget remains poorly understood^10^. To understand the seismic deformation in carbonate rocks, many deformation experiments have been performed in the laboratory on centimeter-size samples^11–16^. These experiments clearly show that extreme comminution, combined with decarbonation, results in massive reduction of friction, further promoting seismic slip^11,12^. Despite recent studies on natural carbonate-hosted faults^17–23^, the scaling of deformation experiments to nature remains problematic because fault planes tend to be geometrically irregular and compositionally heterogeneous.

**Fig. 1:**
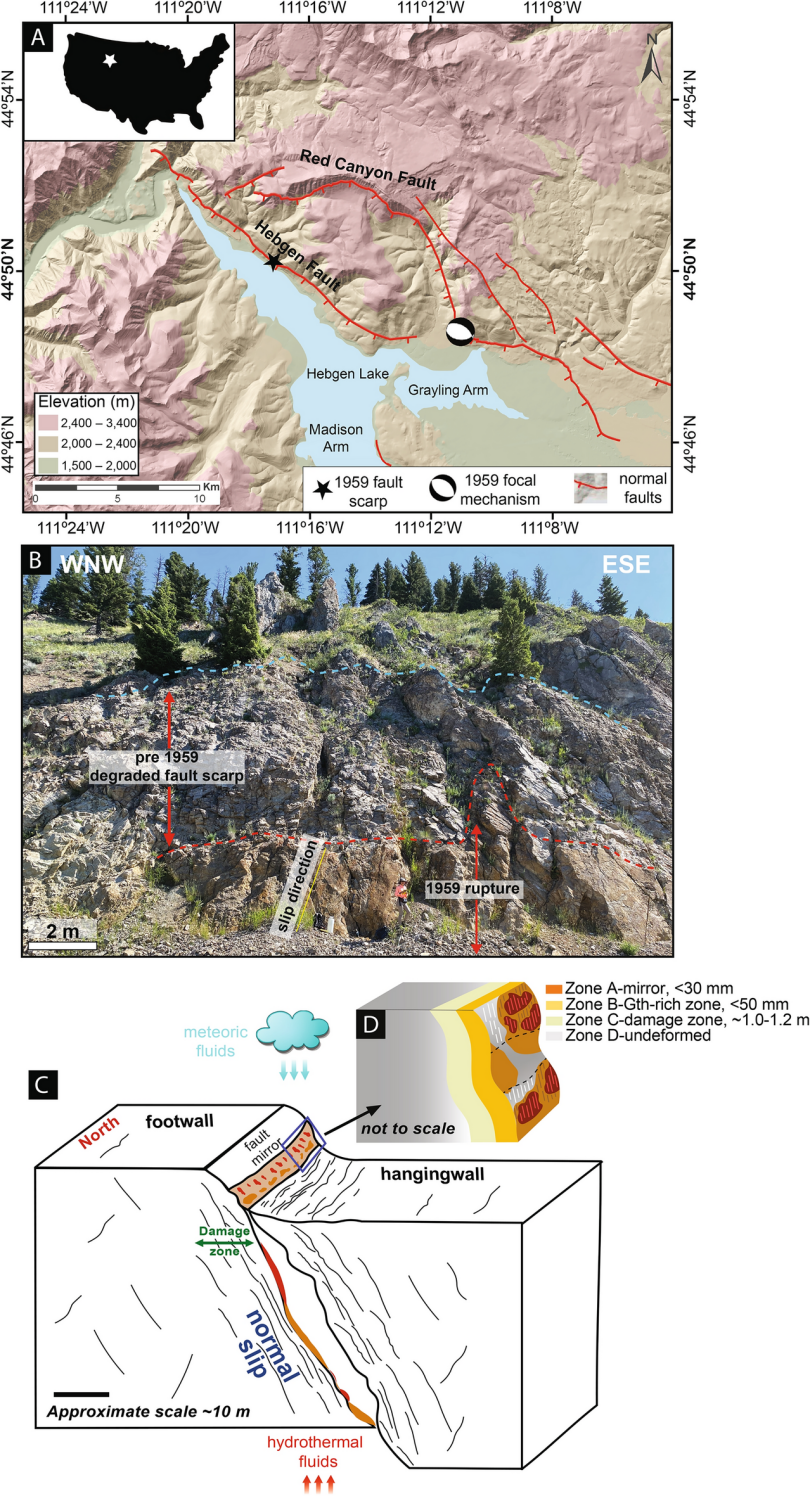
(**A**) Location of the Hebgen Lake Fault, Red Canyon Fault in Western Montana and other normal faults, the S31 Creek bedrock fault scarp, and 1959 main shock focal mechanism^25^. (**B**) Central section of bedrock fault scarp along Highway 278 including the 1959 fault mirror below the red dash line and degraded fault scarp resulting from historic ruptures between the blue and red dash lines; GPS coordinates: 44.834° N, 248.723° E, 2027 m asl (black star on (**A**)). Details on historic fault degradation are in^28^. The fault plane strikes N120° E, dips ~ 66° S and cuts through Cambrian Meagher Limestone. The hanging wall is either not exposed or covered with scree. (**C**) Conceptual block diagram of the Hebgen Lake Fault showing fault mirror and damage zone in the Meagher Limestone. (**D**) Detail of the fault structure with four juxtaposed zones including the undeformed carbonate (**D**), the damage zone (**C**), the goethite botryoidal deposits (**B**) and the fault mirrors (**A**). The Figure was created using Adobe Illustrator v.29.0 and Arc-GIS Pro v.3.2.

To advance our understanding of physico-mineralogical conditions preceding thermal decomposition and frictional behavior of seismogenic carbonate-hosted faults, we target the August 17, 1959, Mw7.2 Hebgen Lake Earthquake in the West Yellowstone area^24^, the largest historic seismic event on a normal fault in the United States. During this double event, the Hebgen Lake Fault (HLF) and the Red Canyon Fault (Fig. 1A) produced a ~ 40 km long surface rupture, with maximum displacement up to 7.7 m, on a surface dipping 60 to 85°, resulting in 28 casualties^24–27^. Overall, the surface rupture zone is covered by unconsolidated Holocene sediment, except at Section 31 Creek (S31C), where a 250 m-long bedrock fault scarp is visible within the Cambrian Meagher Limestone (Fig. 1B,C and Supplementary S1A)^28^. Cosmogenic ^36^Cl on samples from this fault exposure indicate at least 7 earthquake events during the past 24 ka^29^. This remarkable exposure allows us to investigate the details of fault slip processes, such as frictional heating and mineral growth/reactions during seismic rupture along a major carbonate-hosted normal fault. Regional structures and previous seismological investigations show that the 1959 rupture initiated at 12 km-focal depth and propagated through carbonates, all the way to the S31C surface fault scarp^29^.

We analyze microstructures and iron oxide/hydroxide assemblages to decipher the thermal fingerprints of seismic slip produced by the 1959 earthquake. We unravel the fault deformation and peak temperature by combining mineralogical and geochemical analyses, lattice-preferred orientation (LPO), magnetic fabric, and thermomagnetic analyses.

The wide variation of frictional heat, on the Hebgen Lake Fault at the scale of a few meters, suggests that further understanding the mechanical behavior of seismogenic carbonate faults will require field-based analysis, at a scale larger than that of conventional deformation experiments. The new mineral and magnetic criteria discussed in this study to evaluate variations in frictional heat could be applied to other carbonate-hosted seismogenic faults, e.g., in the Apennines of Italy^30^, the Longmenshan Belt of China^31^, the Corinth Rift of Greece^32,33^ or the Zagros Belt of Iran^34,35^.

## Results

The HLF footwall at the S31C locality presents four adjacent zones (Fig. 1B,C): (A) the mirror zone, a ~ 30 mm-thick surface, with glossy patches of either calcite-dolomite (CaCO_3_, CaMg(CO_3_)_2_), goethite (αFeO(OH)) or hematite (αFe_2_O_3_) slickenlines, that cut abruptly through all previous structures; (B) a ~ 50 mm-thick zone with extensive layered-botryoidal goethite; (C) a ~ 1.0–1.2 m-thick yellowish damage zone, with a network of sub-parallel fractures that decrease in density away from the mirror, and (D) the least deformed greyish limestone.

**Fig. 2:**
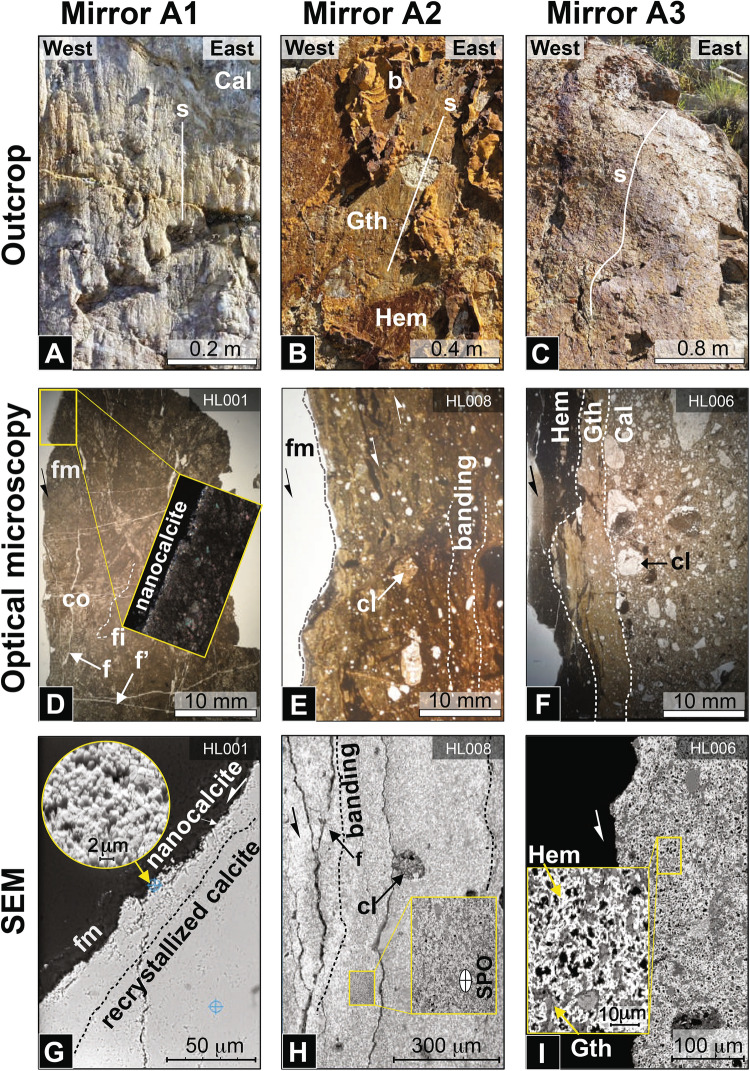
Outcrop-scale fault mirror characteristics of the Hebgen Lake Fault, optical photomicrographs, and Scanning Electron Microscopy (SEM) backscatter images of fault rocks cut perpendicularly to fault mirror (fm) across the three types of fault mirrors (carbonate, goethite and hematite). (**A**) Carbonate fault mirror (A1), carbonate with slickenlines (s). (**B**) Goethite fault mirror (A2), ochre botryoidal goethite (b), ochre goethite slickenlines (g) grading into reddish hematite slickenlines (h), and tension fractures (f). (**C**) Reddish hematite-rich (h) mirror A3 with slickenlines (s); note fault mirror undulations perpendicular to downslip direction. (**D**) HL001—across carbonate mirror, showing juxtaposed coarse (co) and fine (fi)-grained domains, with nanocalcite along the fault mirror and two sets of fractures (f and fʹ); (**E)** HL008—across the goethite mirror, showing carbonate clasts, banding and asymmetric sigmoidal microstructures indicative of local shear; (**F**) HL006 – across carbonate (Cal), goethite (Gth) and hematite (Hem) layers; also note showing the paucity of carbonate clasts in the hematite zone; (**G**) HL001—showing details of the nanocalcite zone along the carbonate fault mirror (fm); inset showing the uniform and equant nanocalcite grain size; (**H)** HL008—across a goethite-rich layer, showing a polycrystalline carbonate clast (cl), banding and shape preferred orientation (SPO) of carbonate and goethite grains; (**I**) HL006—across a A3 hematite-rich layer, showing shape preferred orientation (SPO) of carbonate, goethite and hematite grains. The mid gray tones are goethite (Z ~ 89) while the bright gray tones are hematite (Z ~ 160). The goethite clusters probably resulted from flocculation.

Macroscopically, zone A shows three types of fault mirrors with distinct slickenlines: A1 mirrors with carbonate slickenlines (Fig. 2A); A2 mirrors with localized goethite slickenlines on top of layers of botryoidal goethite (Fig. 2B); and A3 mirrors with pervasive hematite slickenlines (Fig. 2C). Hematite exclusively occurs along the HLF 1959 slip surface as a <  ~ 1 mm-thick red-maroonish slickenline layer on non-striated goethite deposits (Figs. 1B, 2B,C). All hematite patches observed on the HLF are characterized by slickenside fabric and approximately 0.3–0.6 m tall by 1–2 m wide. The hematite fault mirrors occur preferentially on topographic ridges (humps) of the S31C fault scarp (Fig. 2C). All three fault mirrors locally grade into each other with similar slickenline trends and normal sense of shear.

Microstructural details of the A1–A3 fault mirrors are described below (Figs. 1C, 2D–I, Supplementary S2 and Supplementary S3).

Carbonate fault mirrors (A1) are composed of carbonate grains, < 10 μm in diameter (Fig. 2D,G). These grains exhibit equigranular microstructures with high-angle (~ 120°) grain boundaries, consistent with thermal equilibration (Fig. 2G and inset). Euhedral pyrite cubes, ranging in size from ~ 1 to 50 μm, are found locally in aggregates of tens to hundreds of grains, regardless of their distance from the mirror (Figure Supplementary S2). Some diagenetic pyrite grains close to the carbonate fault mirror (A1) exhibit compositional and microstructural heterogeneity, reflected in backscatter electron images. The reflective part of the mirror is comprised of a discontinuous zone approximately 200 μm-thick, composed of fine, equant, calcite grains without preferred crystallographic orientation, referred to as nanocalcite (with a grain size smaller than 1 μm). The systematic presence of topographic depressions (pit) around the pyrite grains indicates that sulfides reacted with oxygen and water, leading to the localized dissolution of carbonate (refer to reactions in Figure Supplementary S2). Similar reactions also resulted in morphological pits on these A1 surfaces. A1 mirrors also exhibit prominent slickenlines, sub-parallel to each other over several cm^2^, and a network of open shear fractures, less than 5 mm wide, sub-parallel to the fault mirror. The density of fractures notably increases towards the fault core. The nanocalcite layer that forms the fault mirror displays equant nanosize, densely packed, equigranular calcite grains (~ 1 μm).

Goethite fault mirrors (A2) are characterized by ochre-colored, macroscopically isotropic, equigranular goethite, calcite, dolomite, and a few subrounded clasts of carbonates, quartz, and rare feldspar, some showing undulose extinction (see Fig. 2E). These clasts are typically < 100 μm in size and slightly elongated parallel to the fault mirror. Goethite masses form botryoidal concretions with asymmetric compositional banding, broadly parallel to the fault mirror, composed of alternating bands of goethite-rich and goethite-poor material (Figure Supplementary S1B). These goethite masses are microcrystalline and individual grains cannot be discerned macroscopically. Goethite fault mirrors (A2) transition into hematite fault mirrors (A3) across a sheared contact zone. Moreover, a network of hematite-filled tension fractures, up to 10 mm wide, cuts through A2 mirrors at high angles to the mirrors. In some areas, the goethite-rich layer displays slickenlines and elongated grains of goethite and calcite, indicating a shape-preferred orientation parallel to the fault mirror (see Fig. 2H).

Hematite fault mirrors (A3) consist of three superimposed layers at increasing distances from the slip surface (see Fig. 2F): *layer Hem*—a hematite-rich domain with a few quartz clasts and no carbonate clasts; in these layer, hematite is microcrystalline and individual grains cannot be distinguished macroscopically, the hematite slickensides appear platy and parallel to the mirror; *layer Gth*—a ~ 3 mm-thick goethite-rich, comminuted domain bearing fewer and smaller subrounded clasts of quartz and carbonates, along with asymmetric lens-shaped domains indicative of shearing; *layer Cal*—a breccia containing abundant, rounded to angular clasts of quartz, feldspar, and recrystallized polycrystalline quartz clasts. Multiple quartz clasts in *layer Cal* present a slight elongation in the same direction, forming a subtle shape-preferred orientation parallel to the fault mirror.

**Fig. 3:**
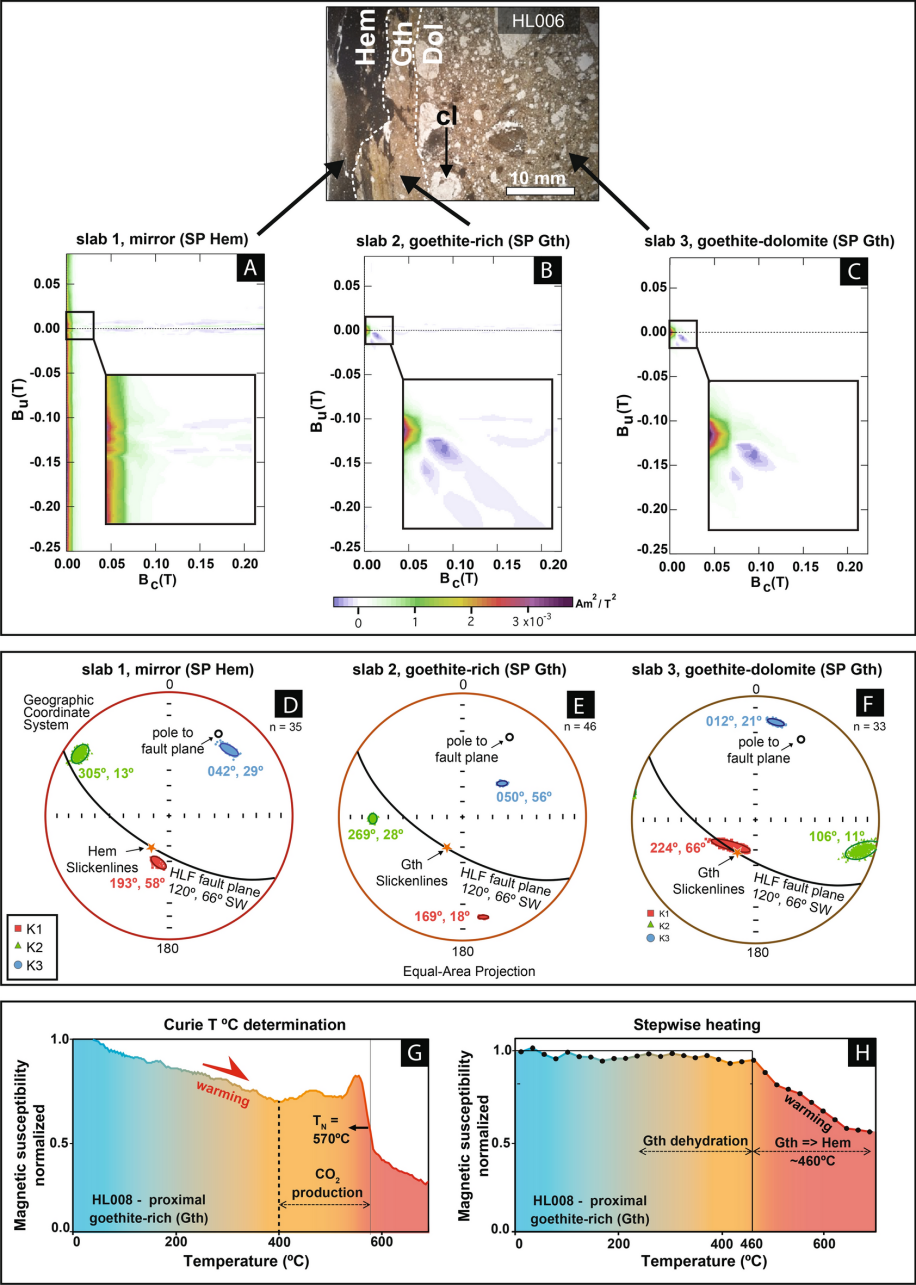
(**A**) First order reversal curve (FORC) diagram of the hematite-rich fault mirror HL008 (layer Hem). (**B**) FORC diagram of proximal goethite-rich (layer Gth). **C**. FORC diagram of distal goethite-rich layer. (**D–F**) Stereonet of bootstrapped AMS principal axes for sample HL011 in the geographic reference framework (for raw data see Supplementary Figure S5)—(**D**) slab 1, hematite-rich fault mirror; (**E**) slab 2, goethite-rich fault mirror; (**F**) slab3, goethite-rich fault mirror. Both bootstrapped and raw directional data are spatially homogeneous and show magnetic fabric consistency at the scale of a few centimeters. For the fault mirror (**F**) in which AMS is controlled by SP hematite, K_3_ axes, poles to magnetic foliation, plot close to the normal to the fault plane (N120°, 66° SW) and K_1_ axes, magnetic lineations (N198°, 58°) plot close to the slickenlines direction (N210°, 66°). (**G**) Thermomagnetic experiment on fault layer 1 (proximal Gth-rich) showing the high Curie temperature (570 °C) related to low-Ti hematite. The magnetic susceptibility decrease up to 400 °C is attributed to goethite dehydration. Between 400 and 570 °C, carbonate breakdown releases CO_2_ which in turn buffers *f*O_2_ and hence resulting in formation of small amounts of maghemite or magnetite and in a slight increase in magnetic susceptibility. At ~ 570 °C, the magnetic susceptibility sharply drops at a Néel temperature consistent with presence of a low-Ti hematite. (**H**) Stepwise flash heating experiment at intervals of 25 °C for 2 min in air atmosphere showing minor decrease in magnetic susceptibility caused by goethite dehydration up to 400 °C followed by transformation of goethite to hematite and decrease in magnetic susceptibility from 460 to 700 °C. Temperatures in the thermomagnetic experiments are determined through thermocouples with an accuracy of 2 °C.

The nanometer grain size of goethite and hematite in the A3 hematite fault mirror is indicated by their superparamagnetic (SP) properties (Fig. 3A–C). This fault mirror hosts diamagnetic (calcite, dolomite, quartz, kaolinite) and antiferromagnetic (goethite, hematite) minerals that have various contributions to magnetic susceptibility (Supplementary—Table S1). To increase the resolution of magnetic analyses, the samples were sliced into three adjacent 3.5 mm-thick slabs parallel to the fault mirror (Supplementary S4). Overall the two antiferromagnetic phases, goethite and hematite, dominate the magnetic susceptibility (ranging 65–100% and 0–40%, respectively). The quantitative contribution of these phases to magnetization is determined through Isothermal Remanent Magnetism (IRM) experiments (Supplementary S5) showing that, in the hematite fault mirror, goethite (mean coercivity, B_h_ ~ 2.94 T) and hematite (B_h_ ~ 2.02 T) account respectively for ~ 91.2% and ~ 8.8% of remanent magnetization.

Grain size distribution of magnetic minerals is determined through First Order Reversal Curve (FORC) analysis, a powerful method to resolve magnetic granulometry^35^. Figure 3A shows a prominent SP fringe along the vertical axis, indicating the presence of SP hematite in slab 1 (~ layer Hem) of hematite fault mirror A3. Conversely, Fig. 3D,E show the presence of a mixture of SP and minor single-domain (SD) goethite assemblage in slabs 2 and 3.

The Anisotropy of Magnetic Susceptibility (AMS) provides information about the magnetocrystalline orientation of goethite and hematite nanograins. The three slabs 1 to 3 show distinct AMS fabric as a function of distance to the fault mirror (Fig. 3D–F and Supplementary S6). Samples from the hematite fault mirror slab 1 (mirror) display a prominent magnetic fabric that broadly coincides with the fault mirror orientation and the slickenlines direction. The degree of anisotropy Pʹ ranges from 1.106 to 1.491, which indicates a ferromagnetic *s.l.* origin and oblate magnetic fabrics (T ~ 0.128 to 0.236) are most likely due to the deformation process and the planar magnetocrystalline anisotropy of goethite and hematite. The electron backscatter diffraction (EBSD) data shows that the AMS-carrying phases goethite and hematite have weak lattice-preferred orientations, ~ 2.5 multiples of uniform distribution (Supplementary S7). Goethite displays maximum point distribution of its (001) and (100) crystallographic axes whereas hematite crystallographic axes distribution (0001) and (11$\overline{2}$0) form partial girdles indicative of partial activation of multiple slip systems.

Two types of thermomagnetic experiments shed light on mineral reactions of the HLF rocks during frictional heating (25–700 °C). In the Curie temperature experiment (Fig. 3G), an HLF goethite sample is continuously heated under argon flux to reduce oxidation. From 25 to 400 °C, the magnetic susceptibility (κ) decreases by ~ 30% due to goethite dehydration, while mass decreases by < 12%. From 400 to 570 °C, we observe minor κ fluctuations attributed to the breakdown of iron-bearing minerals. At ~ 570 °C, κ sharply drops, corresponding to the Néel temperature (T_N_) of a low-Ti hematite.

The experiment becomes irreversible at 570 °C (Figure Supplementary S8), indicating lab-induced magnetite formation. This experiment also confirms that during slip in the natural conditions, in the hematite fault mirror (A3), peak temperature never exceeded 570 °C; otherwise, magnetite would have formed. The stepwise heating experiment (Fig. 3H) involves incremental and cyclic heating, in steps of 25 °C for 2 min, and cooling to room temperature. This 2 min duration of heating is intended to mimic the short-lived^25^ seismic slip conditions at shallow depth. Up to 460 °C, mass-normalized magnetic susceptibility (χ) remains stable. Above 460 °C, calcium carbonate decarbonation and goethite dehydration occur concurrently until ~ T_N_, causing a similar decrease in χ as in the Curie temperature experiment.

To further constrain co-seismic peak temperature along the HLF, we also applied Rock–Eval, a well-established hydrocarbon pyrolysis method used to determine the burial temperature of sediments^37,38^. Based on 2 sets (5 samples each), separated by 0.1 m from each other, the results show a substantial decrease in hydrocarbon contents (S_1_–S_2_–S_3_) by 55 to 30% between 1.5 and 7.5 mm away from the fault mirror (Supplementary S9 and Supplemental Table S2).

## Discussion

### Origin and significance of nanoscale iron hydroxides, oxides and carbonates

The most likely Fe source for the iron oxides and hydroxides along the HLF lies in the diagenetic sulfides of the Meagher Limestone, as shown by the carbonate dissolution pits surrounding sulfide grains (Supplementary S2). Also, close to the fault mirror, pyrite shows a heterogeneous internal structure (Supplementary S2C) indicative of incipient thermal decomposition that typically occurs around ~ 253 °C^39^.

Goethite occurs along the HLF as botryoidal concretions cut by the fault plane (Fig. 2E); these deposits predate the 1959 earthquake. Also, the goethite botryoidal morphology with its typical concentric layers suggests formation in a stagnant aqueous fluid-rich setting^40^, i.e., in between major seismic events and absence of deformation. These very fine grain size deposits (~ 25 nm) are documented primarily by their superparamagnetic behavior shown in the FORC analysis (Fig. 3B,C). In general, goethite forms through chemical weathering of iron oxides and sulfides or precipitation from iron-rich aqueous solutions^41^. In the case of the HLF (Fig. 2B), weathering can be ruled out because these deposits occur only along the fault plane and not elsewhere in the bedrock. Therefore, goethite must have formed through percolation and precipitation of supergene iron-rich solutions related to pyrite alteration (see Sect. 2.3). The goethite magnetic fabric most likely resulted from unidirectional fluid flow deposition, as shown elsewhere by deposits in an industrial pipe filled with precipitated calcite and goethite^42^.

Hematite in the Meagher Limestone has been observed only at the S31C outcrop, along the HLF 1959 fault mirror (A3), as slickenlines forming a <  ~ 3 mm-thick layer, always associated with and directly overlying the goethite-rich layer (Fig. 2B,C). The position of this polished hematite layer atop the fault structure (Fig. 1C), its excellent preservation, and identical slickenside orientation with other slickensides indicate that the hematite slickenlines developed synkinematically during the most recent slip event of the HLF in 1959. ^36^Cl isotopic data^29^ shows that the samples used in our study originate from the 1959 slip surface. Therefore, the striated hematite on this slip surface must have formed through the goethite to hematite dehydration reaction during the 1959 slip event. Also, no hematite was found on the older parts of the HLF produced by previous seismic events, probably because hematite readily hydrates to goethite in the presence of fluids.

At the microscopic scale, the coseismic dehydration of goethite into hematite is documented by the hematite overgrowths surrounding goethite grains (see enlarged inset of Fig. 2I), further attesting to coseismic frictional heating along the HLF slip surface. Several studies on reddish, hematite slickenlines on carbonate fault mirrors elsewhere concur that they formed under ~ 300 °C and at seismic slip velocities^43–46^. Further, in heating experiments without deformation, goethite reacts to hematite between 200 and 300 °C^47–49^, which supports our interpretation that hematite formed through coseismic frictional heating because localized, elevated temperatures are unique to seismic slip. The difference between our results on carbonate rocks and experiments on pure natural goethite lies in the delay in oxidation of goethite into hematite caused by CO_2_ release during decarbonation. We acknowledge, also, that this reaction is grain-size dependent^50^, and to test the thermal stability of goethite upon heating, we conducted thermomagnetic experiments to simulate coseismic frictional flash heating on the HLF slip surface. In our experiments, the dehydration reaction began at ~ 100 °C and was completed at ~ 460 °C, while hematite continued to form up to 620 °C (Fig. 2A,B). Combined with X-ray diffraction analyses (Supplementary Table S1 and Figure S10), these experiments support goethite dehydration to hematite upon frictional heating (Supplementary Eq. 4). Furthermore, the quasi-parallel direction of the AMS magnetic lineation within the hematite fault mirror and the hematite slickenlines (Fig. 3D) support their shared coseismic origin.

In previous carbonate deformation experiments, nanoscale calcite typically forms glossy surfaces similar to natural fault mirrors. These surfaces may also bear nanocalcite that forms through decarbonation and subsequent recrystallization of portlandite^11–13,15,46^ or, more seldomly, through extreme comminution and sintering^44^. On the HLF slip surface, carbonate slickensides occur on a few discontinuous and irregular patches, a few tens of centimeters across (Fig. 2A). The most glossy of these patches preserve a thin nano-calcite layer, hence forming a fault mirror (Fig. 2D,G). We interpret this nanocalcite layer as resulting from thermal decomposition of carbonates based on the comparison between the HLF A1 mirror microstructures in this layer with microstructures proven elsewhere to result from either decarbonation or from simple grain size reduction: (1) cataclastic comminution follows an asymmetric power-law crystal size distribution (CSD), which is clearly not the case at the HLF (Figure S3A–C); (2) grain size reduction through brittle fracturing and mechanical grinding produces rounded grains^44^^Figure 2E,F^, which is not the case at HLF (Figure S3); (3) as decarbonation takes place at temperatures ~ 400–600 °C, the recrystallized grains tend to develop equant, sintered microstructures^15^^Figure 5D,^^16^^Figure 13B^ which is precisely what we observe on the A1 mirror at HLF (Figure S3A).

The XRD data (Supplemental Table S1 and Figure S10) shows a drastic increase in hematite concentration in the A3 fault mirror. Overall, three different fault mirrors (carbonate, goethite, and hematite) show that post-seismic mineral assemblage on the slip surface is controlled by the heterogeneous distribution of mineral precipitates along the fault domain. Also, the lack of plastic or cataclastic strain in the goethite botryoidal concretions shows that they formed during interseismic periods of the HLF history when the fault experienced limited to no creep between seismic events. Further, our observations show that previously precipitated goethite concretions were coseismically smeared into hematite slickenlines (Fig. 2B,C) due to dynamic frictional heating. Due to morphological undulation along the fault plane, goethite was unevenly sheared into slickenlines in some places but not sufficiently heated to form hematite (Fig. 2B). The patchy distribution of hematite slickenlines also suggests that frictional heat was not evenly distributed along the slip surface.

### Contribution of AMS to fabric development and deformation history

We calculate the diamagnetic (calcite, quartz, dolomite, kaolinite) and antiferromagnetic (goethite, hematite) contributions to magnetic susceptibility using published intrinsic susceptibilities and XRD modal percentages measured on the hematite fault mirror (Table S1; Figure S5). These calculations highlight goethite’s dominant contribution (65–100%) to magnetic susceptibility across all slabs and hematite’s major role (~ 40%) in the fault mirror slab.

The magnetic grain size of goethite and hematite, evaluated using magnetic hysteresis and FORC data, corresponds to superparamagnetic (SP) domain state (Fig. 3C–E), i.e., nanoscale sizes of ~ 30 nm for hematite^36^ and ~ 25 nm for goethite^51,52^. Based on IRM acquisition experiments, goethite and hematite, respectively, account for ~ 91% and ~ 9% of magnetic remanence (Supplementary S5).

**Fig. 4:**
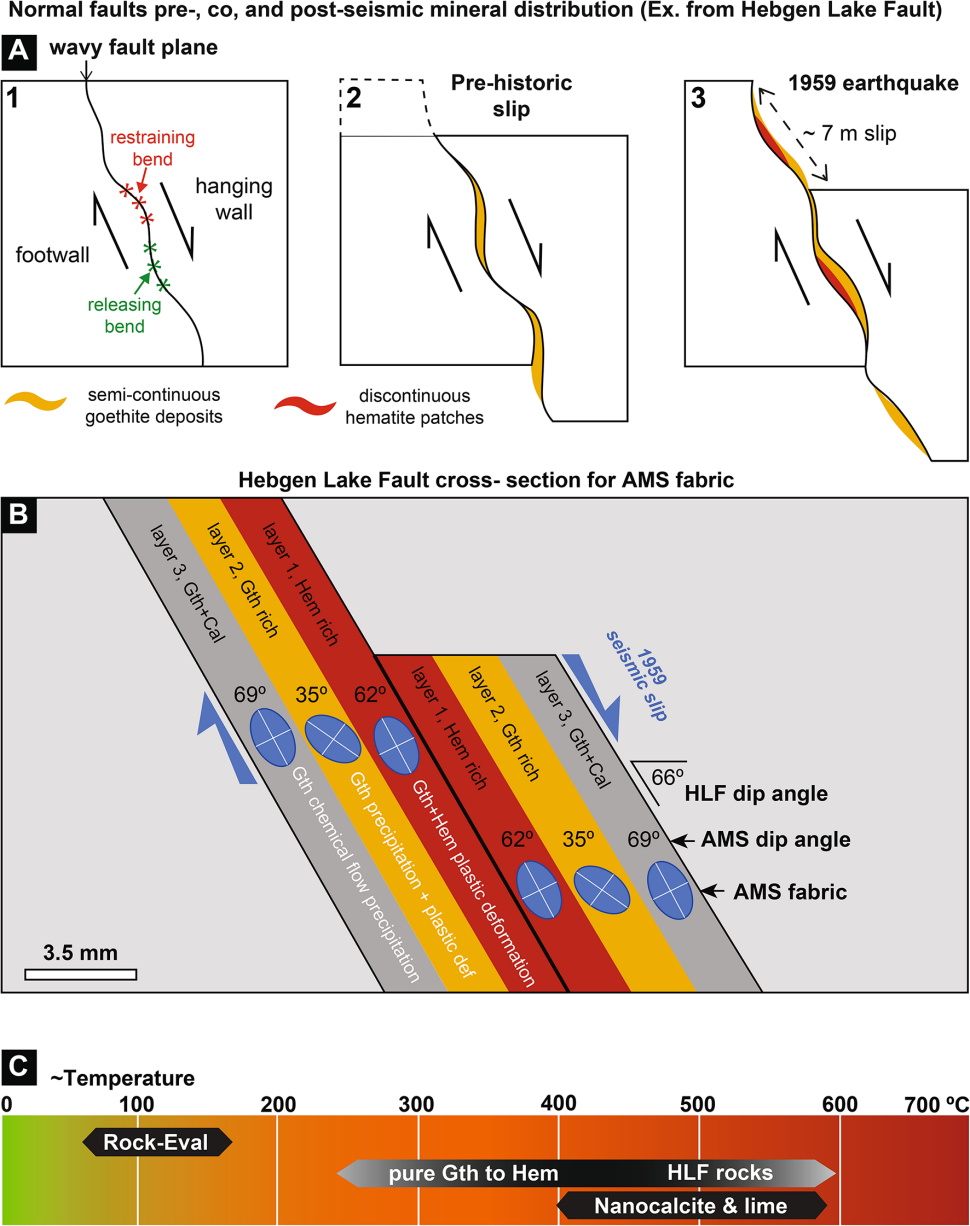
(**A**) Schematic model showing the evolution of the Hebgen Lake Fault through pre-seismic (1), interseismic (2) and co-seismic deformation steps. (**B**) AMS fabric model for the Hebgen Lake Fault showing a symmetrical structural zonation for the hematite-rich fault mirror (A3). The oblate symmetry of the AMS in this material (Supplementary Figure S6) also supports precipitation in a planar conduit. Since goethite developed slickenlines in mirror A2 and these mirrors grade into hematite-rich mirrors (A3), and most likely shear deformation of goethite affected slab 2 as well. The AMS K_1_ direction (magnetic lineation) in slab 3 is interpreted to represent quasi-exclusively the direction of fluid flow, *per descensum*, along the dip direction of the HLF plane. In contrast, in slab 2, the pre-existing downdip fluid flow direction recorded by AMS is modified by shear deformation, resulting in a shallower plunging K_1_ direction and shallower dipping magnetic foliation (plane to K_3_ axis). (**C**) Summary of thermo-chemical constraints of frictional heating (0–700 °C). The left side of the middle arrow (pure Gth to Hem) represents temperatures from experiments on pure goethite (see text for details) whereas the right side of this arrow represents the HLF rock thermomagnetic experiments (Fig. 3G,H).

In general, the AMS of deformed rocks arises from the combined magnetostatic, magnetocrystalline, and interaction anisotropies of minerals^53,54^. In the HLF rocks, the AMS arises mainly from the strong magnetocrystalline anisotropy of goethite and hematite (Supplementary Table S1), as shown elsewhere^53–55^. For SD and SP goethite, the AMS tensor mimics the grain SPO, unlike in the case of MD grains that have an inverse fabric^39,56^. Similarly, SP hematite shows a normal AMS fabric^41,55^. Therefore, the AMS of goethite-rich and hematite-rich HLF rocks reflects the mineral SPO (Figs. 2I, 3D–F). As goethite is a hydrous mineral deposited by fluids, its fabric must have been produced through fluid precipitation-percolation, primarily without much plastic deformation, i.e., in interseismic periods (Fig. 4A,B). Moreover, the AMS long axis reflects the unidirectional fluid flow direction, as shown elsewhere by^42^ in hydrothermal deposits. The source of the HLF ferruginous fluids will be discussed in a subsequent study. The presence of slickenlines on goethite fault mirrors (A2) that seamlessly transition into hematite fault mirrors (A3) indicates that localized shear deformation of goethite, due to frictional sliding below 300 °C, likely took place in slab 2.

In slab 3, the AMS K_1_ direction (magnetic lineation) represents quasi-exclusively the direction of fluid flow along the dip direction of the HLF plane (Fig. 3D–F and Supplementary S4) as shown elsewhere by^42^. In contrast, in slab 2, the pre-existing AMS is modified by shear deformation, resulting in a shallower plunging K_1_ trend and dipping magnetic foliation (plane normal to K_3_ axis). In slab 1, fault mirror, the AMS partially arises from the weak SPO of hematite and goethite clusters (Fig. 2I). The distinctively lower degree of anisotropy in slab 1 compared to slabs 2 and 3 is explained by the lower intrinsic anisotropy of hematite compared to goethite^41,57^. We interpret the hematite microfabric in the fault mirror to result from synkinematic comminution, under moderate temperature (150-350 °C), and shear deformation of the underlying layer of precipitated SP goethite. The AMS K_1_ direction matches the slip direction along the hematite fault mirror.

### Implications of fault mirror development for the Hebgen Lake Fault

The Plio-Pleistocene slip history of the Hebgen Lake Fault, previously established by^29^ using chlorine isotopes on the S31C scarp, comprised up to six events at ~ 23.8, 20.3, 7.0, 2.6, 1.7, and 0.4 ka ago, prior to the 1959 Mw7.2 event^58^ confirmed these results using Lidar and Schmidt hammer methods, although conventional paleoseismic trenches documented only three main events^26^. Finally, surveys performed immediately after the Mw 7.2 earthquake^59^ showed that the S31C fault scarp resulted from the 1959 slip event. This young age also partially accounts for the sporadic preservation of a nanocalcite thin layer on the A1 carbonate fault mirror that we report here for the first time (Fig. 2D,G).

In the following, we first briefly review prevailing conditions for fault mirror development and then, in this context, discuss the temperatures inferred from the carbonate-, goethite- and hematite-mirrors specifically for the HLF.

Carbonate fault mirrors are naturally glossy and smooth surfaces formed during slip at subseismic to seismic velocities^43,60^. These thin zones consist of layers of nanoscale grains^43,44,60,61^. Furthermore, deformation experiments show that fault mirrors result from microstructural (e.g., comminution) and chemical processes (e.g., decarbonation) resulting in dynamic weakening^44,45,60,62^, and therefore, this is a critical process to understand earthquake mechanics. We however acknowledge that decarbonation does not systematically occur along carbonate seismogenic faults although this process is well documented elsewhere^18–20^. The continuous formation and destruction of fault mirrors throughout seismic slip generally leads to their patchy occurrence on a fault plane^44^. Well-preserved glossy mirrors in carbonate faults have been attributed to the sintering of nanocalcite grains^50,63,64^.

The HLF carbonate fault mirror (A1, Fig. 2A) exhibits evidence of frictional heating, which we estimate through Rock–Eval experiments by observing a significant reduction in hydrocarbon contents (S_1_, S_2_, S_3_) ranging from 55 to 30% over a distance of 1.5–7.5 mm from the fault mirror (Supplementary Figure S9B). This decrease in hydrocarbon content is most evident in the S_1_ parameter, which primarily reflects the release of methane (CH₄) at a minimum temperature range of 300–350 °C. Our analysis detected this release at a distance of 6.5 mm from the fault mirror. In contrast, under natural conditions in carbonates with the presence of O₂, the peak of katagenesis typically occurs around 120 °C^65^ (Supplementary Figure S9A). However, in our pyrolysis experiments, conducted in a helium atmosphere to avoid oxidation, the peak katagenetic temperature occurred between 345 and 420 °C. Therefore, it is likely that under the shallow, oxidizing conditions of the Hebgen Lake Fault, closer to the surface, the natural peak temperature for frictional heating pyrolysis would have been around 120 °C. Using this temperature, we determine the bulk katagenesis temperature as ~ 120 °C at 4.5 mm from the slip surface, a value in agreement with our numerical modeling results (Supplementary Figure S9B and Fig. 4C). The stability of kaolinite in sample HL011S1C56 (Table S1) also places an upper limit on the maximum temperature reached during slip to ~ 400 °C^66^.

Further, the uniform grain size and equant shape of nanocalcite grains (~ 1 μm) along the carbonate fault mirror (Fig. 2G inset and Supplementary S3A) most likely did not form through comminution because this process typically forms broad crystal size distributions (CSD)^67^. Instead, the nanocalcite microstructure resulted from either from superplastic flow^44^ or from decarbonation, a thermally and chemical-diffusion driven process^68^. Also, the well-defined Ca Ka peak on EDS spectra of nanocalcite indicates high degree of crystallinity (Supplemental Figure S3A inset). We argue that the equant shape of the nanocalcite supports post-kinematic, static recrystallization of lime or portlandite whereas synkinematic superplastic flow and comminution would have resulted in slightly elongated grains. We attribute the weak lattice-preferred orientation of nanocalcite (MUD ~ 2; Supplementary S7) to static recrystallization in a thermal gradient.

In addition, the numerical modelling of frictional heating (Table 1), Rock–Eval results (Supplementary S9), and microstructures indicative of pyrite thermal decomposition (Supplementary S2C), show that coseismic frictional heating took place and most likely affected a ~ 60 mm-thick zone along the fault mirror. Also, the formation of lime and portlandite along slip surfaces is a short-lived process and these decarbonation-related phases tend to be preserved only for a short duration^69^. As the earthquake took place in 1959, these minerals disappeared rapidly through weathering and fault scarp degradation.

**Table 1:**
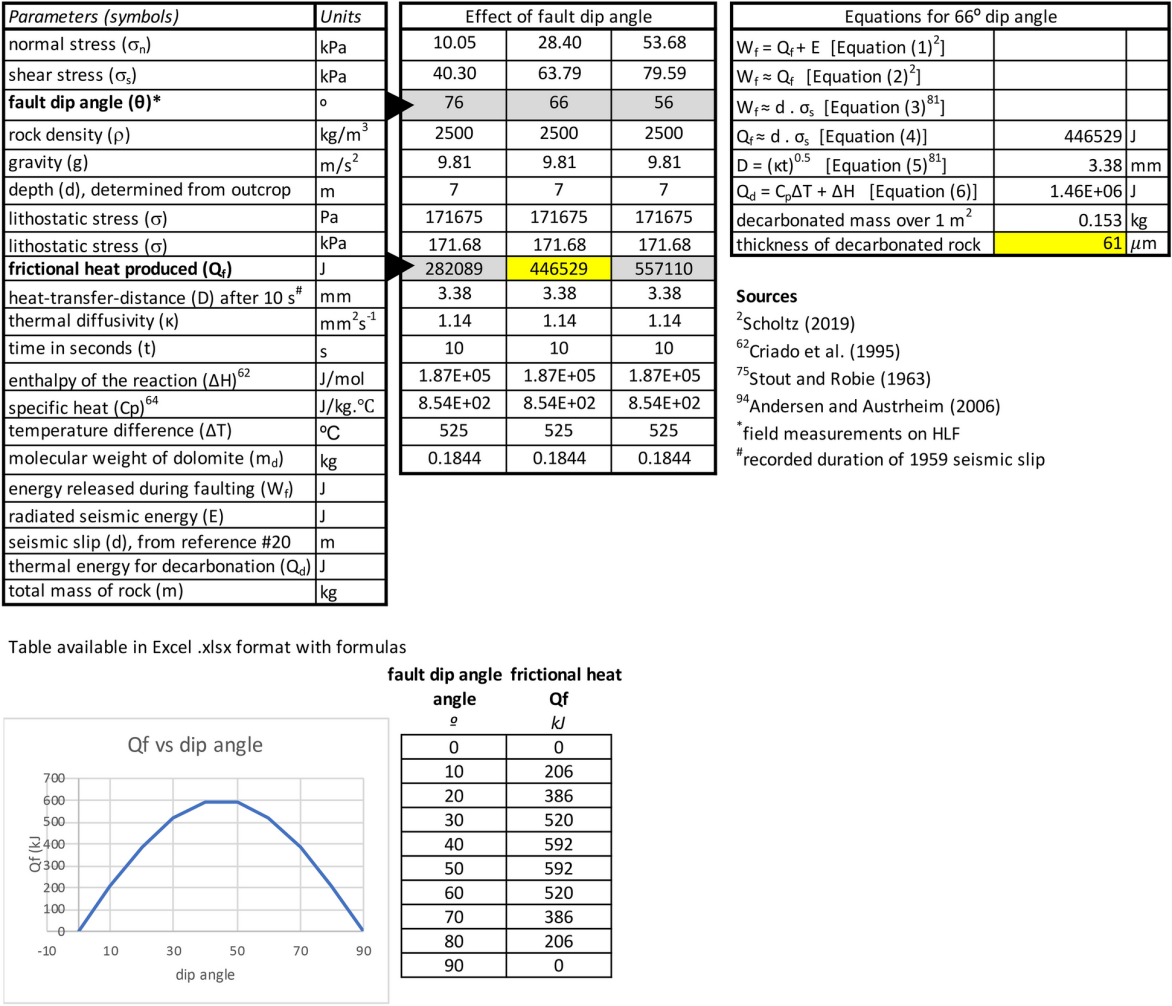
Calculation of frictional heat along the Hebgen Lake Fault plane and plot showing the variation of frictional heat (Q_f_ ) with dip angle.

The A2 HLF goethite mirror (Fig. 2B) consists of a thin layer of slickenside grains (< 50 μm) structurally on top of a layer of botryoidal goethite (< 50 mm) in which the concentric growth layers are visible. The slickenside layer exhibits two types of microstructures similar to those described elsewhere on other carbonate fault mirrors^70^: (1) the sigmoidal microstructures (Fig. 2E) that indicate high normal stress and moderate velocity (~ 100 MPa; ~ 10^–2^ m/s), (2) the mirror-parallel fractures (Fig. 2H) that indicate lower normal stress and higher slip velocities (~ 10 MPa; ~ 0.3 m/s). The range of physical conditions under which these microstructures developed suggests that this mirror recorded seismic slip from the early acceleration phase through the rise of frictional heat. The microstructures above occur in domains parallel to the mirror (Fig. 2E).

The A3 HLF hematite fault mirror (Fig. 2C), based on FORC analysis (Fig. 3A), consists of nanograins of hematite, a mineral with a normal AMS. The hematite mirror bears a magnetic foliation (plane normal to K_3_ axis) close to the fault slip surface and a magnetic lineation (K_1_ axis) quasi identical to the hematite slickenlines (Fig. 3D). Since the thermomagnetic experiments indicate that hematite forms above 460 °C (Fig. 3H), we conclude that the hematite magnetocrystalline anisotropy (Fig. 2D) was acquired as a result of hematite shear deformation under moderate temperature, as shown elsewhere by^71^. This interpretation is consistent with currently available deformation mechanism maps for hematite^72^ and with the partial girdle of hematite crystallographic axes (Supplemental Material—Figure S70).

### Numerical quantification of HLF coseismic frictional heat during the 1959 slip event

We calculate the thickness of host-rock that underwent decarbonation, using fault displacement, dip angle, depth, thermal diffusivity, specific heat, specific mass of limestone, enthalpy and temperature of decarbonation, while considering adiabatic heat transfer after 10 s of slip (Table 1).

The 1959 earthquake produced displacements greater than 7 m in multiple sections of the Red Canyon Fault^27^ and the Hebgen Lake Fault, including at the S31C site (^29^ and this study). We use this approximate 7 m displacement and the estimated depth of the outcrop (~ 7 m) to calculate several faulting parameters for the S31C locality, where the HLF is best exposed.

The energy released during faulting (W_f_) is mostly dissipated as frictional heat (Q_f_) and, to a lesser extent, as radiated seismic energy (E) (2).

1 $${\text{W}}_{{\text{f}}} = {\text{Q}}_{{\text{f}}} + {\text{E}}$$

Indeed, experiments and seismological studies (2) show that radiated seismic energy (E) is small (< 5%) compared with the total earthquake energy (W), therefore;

2 $${\text{W}}_{{\text{f}}} \approx {\text{Q}}_{{\text{f}}}$$

It follows that, per unit area,

3 $${\text{W}}_{{\text{f}}} \approx {\text{d}} \times \sigma_{{\text{s}}}$$

where d is the true displacement, and σ_s_ is the shear stress at the deformation time.

Using the fault plane dip angle θ = 66°, the fault parameters of Table 1, and Eq. (3), we calculate shear stress acting upon the HLF plane at 7 m depth, as σ_n_ = 63.8 × 10^3^ Pa, and frictional heat

4 $${\text{Q}}_{{\text{f}}} \approx {446}.{6} \times {1}0^{{3}} \;{\text{N/m}} = {446}.{6} \times {1}0^{{3}} \;{\text{J/m}}^{{2}}$$

Table 1 also shows the prominent role played by fault plane dip angle (θ) in frictional heat production across a 20° range of dip angles (from 56 to 76°). Equation 4 (Table 1) further highlights the dependency of frictional heat (Q_f_) to shear stress (σ_s_) and, as σ_s_ varies with dip angle [σ_s_ = (σ sin2θ)/2], it follows that Q_f_ varies with dip angle (θ) (see graph in Table 1). In the following, we use the 66° angle as the median value at the S31C locality. This thermal energy produced by friction during a single seismic slip (Q_f_) event is mostly adiabatic because the heat-transfer-distance (D) into the host-rock is minimal during a short-lived (~ 10 s) seismic slip event:

5 $${\text{D}} = (kt)^{{0.{5}}}$$

With thermal diffusivity for limestone *κ* = 1.14 mm^2^ s^−1^ ^73^ and t, time in seconds, we calculate D = (1.14 mm^2^ s^−1^ × 10 s)^0.5^ ~ 3.38 mm after 10 s of seismic slip. This low D value agrees with the ~ 3 mm-thickness of the goethite to hematite reaction zone that we interpret to result from coseismic frictional heat.

Then, we calculate how coseismic frictional heat was used to heat and decompose the host carbonate on both sides of the slip plane (hanging wall and footwall). The thermal energy Q_d_ needed to both heat and decompose a dolomitic carbonate rock of one mass unit is

6 $${\text{Q}}_{{\text{d}}} = {\text{C}}_{{\text{p}}} \Delta {\text{T}} + \Delta {\text{H}}$$

With Cp: specific heat, ΔT: the temperature difference between ambient and decomposition temperature, and ΔH is the enthalpy of the decarbonation reaction. We use the enthalpy ΔH of the endothermic decarbonation reaction from^74^:

7 $$\begin{aligned} & {\text{Ca}},{\text{Mg}}\left( {{\text{CO}}_{{3}} } \right)_{{2}} \leftrightarrow {\text{CaCO}}_{{3}} + {\text{MgO}} + {\text{CO}}_{{2}} \quad \Delta {\text{H}} = {187} \times {1}0^{{3}} \;{\text{J/mol}} \\ & {184}.{\text{4 g}} \leftrightarrow {1}00.{\text{1 g}} + {4}0.{\text{3 g}} + {44}.0{\text{ g}} \\ \end{aligned}$$

Decarbonation of dolomite occurs at temperatures as low as ~ 550 °C^75^ and the host rock was at the time of seismic slip at a temperature of ~ 25 °C, therefore ΔT = 550 − 25 = 525 °C. For dolomite Cp = 0.854 × 10^3^ J/(kg ℃)^76^.

The heat needed to increase the temperature of 1 kg of dolomite from 25 to 550 °C is

$${\text{Cp}}\Delta {\text{T}} = 0.{854} \times {1}0^{{3}} \times {525} \approx {447} \times {1}0^{{3}} \;{\text{J}}.$$

In addition, to decarbonate 1 kg of dolomite, the heat needed is

$$\Delta {\text{H}} \times {\text{m}} = \left( {{1}000/{184}.{4}} \right) \times {187} \times {1}0^{{3}} \;{\text{J}} = {1}0{14} \times {1}0^{{3}} \;{\text{J}}$$

where m = moles of dolomite.

Overall, the heat needed to heat and decarbonate 1 kg of dolomite from 25 to 550 °C is

8 $${\text{Q}}_{{\text{d}}} = \left( {{447} + {1}0{14}} \right) \times {1}0^{{3}} = {1461} \times {1}0^{{3}} \;{\text{J}}$$

The total frictional heat produced by seismic slip over 1 m^2^ fault surface (446.6 × 10^3^ J) must be distributed evenly between the hanging wall and the footwall, hence, the total mass of dolomite that can be decarbonated is m = 446.6/1462/2 kg = 0.153 kg. With a 1 m^2^ surface and a dolomite specific mass of 2500 kg/m^3^, the volume of decarbonated dolomite is v = 0.153/2500 ≈ 61.2 × 10^−6^ m^3^, which corresponds to a maximum decarbonated thickness *t* of ~ 61 μm.

This maximum calculated thickness of Meagher Limestone that underwent decarbonation on one side of the fault is in broad agreement with our microstructural observations of a thin layer of nanocalcite (~ 30 μm). This calculated thickness is most likely overestimated because as previously shown elsewhere^11,42^, dynamic weakening is likely to occur due to grain size reduction, thermal pressurization and coseismic decarbonation. Similarly, we consider the initial coefficient of friction (μ) at the onset of slip (Table 1) and we acknowledge that μ will decrease during slip towards dynamic steady state value of ~ 0.2^11^. When μ falls to its lower value of 0.2, the predicted thickness of decarbonated material falls to ~ 5 μm.

In addition, the uneven attitude and heterogeneity of the fault plane (Figs. 1 and 2) most likely resulted in uneven frictional heat distribution. The calculations above indicate that frictional heat at a distance greater than ~ 1 mm from the slip plane should not affect microstructures such as calcite twins. Finally, the Rock–Eval results constrain the width of the thermal aureole where hydrocarbons were heated by friction (~ 120 °C) along the HLF to 4.5 mm from the slip surface (Figure S7).

### Heterogeneity of coseismic frictional heat and implications for fault behavior

In summary, despite differences in protolith composition, the slickenlines maintain consistent orientation across mirror types (carbonate, goethite, and hematite), which shows that they likely formed under similar dynamic stress states and slip rates, although presumably under different temperatures. The carbonate mirror consists of a thin layer of nanocalcite (< 1 mm-thick) parallel to the fault mirror that is responsible for the mirror’s glossy appearance. Decarbonation above 400 °C documented in the Curie experiment (Fig. 3G), in addition to the uniform nano-calcite grain size (Supplementary Figure S7E), supports a thermally driven reaction for this layer instead of a mechanical wear origin^77^. Our temperature estimate is in excellent agreement with previous experiments^16^, showing that decarbonation in dolomite begins at temperatures lower than previously thought.

Further, previous experiments on natural goethite showed that it dehydrates upon heating from 25 to 300 °C^50,78^. The step-wise heating experiment on the HLF rocks attests that goethite dehydration occurs between ~ 250 and 460 °C (Fig. 3H). Hence the goethite fault mirror most likely records a broad range of frictional temperatures from 25 to 460 °C. In previous heating experiments, the observed temperature of goethite to hematite reaction ranges between 200 and 300 °C^47–49^. In our stepwise heating experiments on the HLF rocks, conducted statically without deformation, we observe that hematite forms above 460 °C (Fig. 3H), and therefore, we interpret the frictional heat associated with the 1959 slip event and the formation of the hematite fault mirror to be also above 460 °C. However, we acknowledge that this reaction is grain-size dependent^50^.

The fault core before the 1959 seismic slip (~ 7 m length) comprised various minerals, including dolomite and goethite, leading to likely variations in core strength along the HLF slip plane due to distinct frictional properties. While these differences may account for variations in frictional heat, the variations in dip angle would majorly impact frictional heat, as highlighted in Table 1, underscoring its importance over other factors such as for example slip zone thickness.

Recent studies have shown the effects of fault geometry/long-wavelength fault surface topography on slip behavior^79–81^. These geometric variations of fault attitude would also determine heterogeneous frictional heat generated along a fault^82^.

In the case of the Hebgen Lake Fault, our assessment of frictional heating variations is primarily based on presence or absence of hematite formed on top of a precursor layer of goethite. The reasons why parts of the HLF do not bear goethite is beyond the scope of this study. The goethite to hematite reaction, documented in the HLF through macroscopic field observations (Fig. 2B), microstructures (Fig. 2F), and thermomagnetic experiments (Fig. 3A,B), constrains frictional heat along the hematite fault mirror (A3). The fact that in the Meagher Limestone, hematite occurs only in patches of the S31C fault scarp (Figs. 1B, 2C,F,I), forming a < 1 mm-thick slickenside layer, adjacent to goethite slickenside patches, shows that frictional heat was heterogeneously distributed. Moreover, we interpret the three types of mirrors at the HLF to have formed during the 1959 seismic slip event. In addition, the AMS fabric acquired by the hematite fault mirror coincides with slickensides and is further testimony of its seismic origin at temperatures > 460 °C. Ultimately, the structural macroscopic record of previous seismic slip events^29^ has most likely been overprinted or degraded to various degrees^58^.

Our results introduce a significant advance in thermal constraints on carbonate fault frictional heat: the goethite to hematite reaction at around ~ 460 °C as a new indicator of seismic activity along carbonate fault planes. Unlike previous markers, such as nanocalcite and portlandite^45,46^, which are transient, this new reaction offers lasting mineralogical evidence. Understanding these processes is crucial as they profoundly reduce friction along carbonate faults, further promoting seismic slip through lubrication by both comminution and decarbonation^11,12,14,15^, and pore fluid pressure increase through CO_2_ production^16^, further promoting failure.

Our understanding of seismic deformation in carbonates has greatly benefitted from friction experiments usually performed at the centimeter scale. Nonetheless, field observation-based investigations remain crucial in developing novel concept-based models. Moreover, the variations in fault dip angle play a critical role in frictional heating and decarbonation along natural seismogenic faults. Investigating and detecting the changes in friction, temperature, and mineral assemblage thus becomes critical instead of assuming a uniform maximum temperature from constant friction. Here, we show that iron hydroxide deposits, formed prior to seismic rupture during interseismic periods in carbonates, constitute valuable frictional heat markers. Our investigations of the Hebgen Lake Fault S31C scarp show that, before the 1959 earthquake, aqueous fluid flowed through fractured rock, chemically altered diagenetic iron sulfides, liberated iron, and resulted in substantial precipitation of botryoidal goethite along the fault plane. The origin of these fluids, meteoric or hydrothermal, is still under investigation. This goethite is then locally heated and reacts to hematite under well-documented thermal conditions. Also, the lowest coseismic frictional marker determined along the HLF consists in the Rock–Eval 120 °C temperature measured at ~ 4.5 mm from the slip plane on the carbonate A1 fault mirror. The second lowest record of frictional heat is indicated by the microstructural heterogeneity of diagenetic pyrite grains close to the slip plane at temperatures *ca*. 250 °C. Further, the presence of a thin nano-calcite layer at the surface of the carbonate fault mirror and the paucity of calcite clasts in the hematite fault mirror attest to carbonate thermal decomposition at temperatures > 400 °C. Finally, frictional heating > 460 °C is recorded along the fault plane through the decomposition of goethite to hematite, which constitutes a powerful new indicator of coseismic frictional heat. Our outcrop-based study of the Hebgen Lake Fault reveals that frictional heating, followed by decarbonation, took place at least locally during the 1959 Mw7.2 earthquake and highlights the need to advance our understanding of rupture processes in carbonate from hypocenter depth to the surface. This study highlights magnetic minerals that had previously been overlooked, which serve as important indicators of fault peak temperature in carbonate rocks.

## Supplementary Information

Supplementary Information 1.

Supplementary Information 2.

Supplementary Information 3.

## Data Availability

All original magnetic, XRD, SEM, EBSD and microstructural data are available at the Zenodo data repository (https://doi.org/10.5281/zenodo.14541699).
