# Supplementary material for: Unraveling carbonate fault dynamics, from friction to decarbonation, through the 1959 Mw 7.2 earthquake in Montana
Source: Sci Rep. 2025 Mar 13;15:8625. doi: 10.1038/s41598-025-89071-4 (PMC11903962; doi:10.1038/s41598-025-89071-4)
Supplement: Supplementary file 1 — Supplementary Information 1. [file 41598_2025_89071_MOESM1_ESM.docx]

­­­Supplementary Materials for

**Unraveling carbonate fault dynamics, from friction to decarbonation, through the 1959 Mw 7.2 earthquake in Montana**

Nina Zamani et al.

*Corresponding author. Email: [nzamania@cougarnet.uh.edu](mailto:nzamania@cougarnet.uh.edu)

**This PDF file includes:**

Supplementary Text

Figures S1 to S9

Table S1

Table S2

References (# to #)

Supplementary Text

Methods

*Field observations* – Samples were collected at the surface of rock exposures. To establish orientation, we used a Brunton magnetic compass with an accuracy of ±1º with respect to magnetic North. Outcrop location, determined using a handheld *Garmin* Global Positioning System, was 44.834ºN, 248.723ºE, 2027 m asl. The outcrop documentation and macroscopic sample details were captured using a *Nikon* D80 digital camera with a macro lens.

O*ptical microscopy* – Samples were systematically impregnated with epoxy prior to cutting to preserve all fine scale details of the fault mirrors. Petrographic thin-sections, with a thickness of 30 μm, were cut perpendicularly to the fault mirror from oriented rock billets and polished by *Texas Petrographics* in Houston, TX. Then, these sections were examined in transmitted and reflected light using a *Nikon* Eclipse LV100NPOL polarizing petrographic microscope.

*Electron microscopy* – High-resolution images were obtained using a desktop *Phenom* G2 XL scanning electron microscope. The microscope operated in scanning- and backscatter-electron modes, with magnifications reaching up to X10,000 and an accelerating voltage of 10 kV. Spot chemical analyses were conducted using energy dispersive spectroscopy (EDS) mode.

*Electron Backscatter Diffraction (EBSD) –* Petrographic observations and FORC analyses indicate that goethite and hematite grains are small (25-30 nm) and, therefore, below the detection limit of conventional EBSD. The analyses were performed using the scanning electron microscope (SEM**) CamScan Crystal Probe X500FE** at Geosciences Montpellier, Université de Montpellier, France. Before EBSD analyses, the thin-sections were polished with 3, 1, and 0.3 μm size diamond paste, and finished with SYTON colloidal silica gel. The accelerating voltage of SEM was 20 kV, with a working distance of 25 mm. Diffraction patterns of the mineral phases were detected on a phosphor screen (located in front of the 70° tilted thin-section in the sample chamber of SEM), and images were acquired through a high-resolution CMOS camera. EBSD data from three HLF samples, with scanning step-size of 0.2 μm, were collected automatically using rectangular grids. Data indexing was done using Aztec software from Oxford Instruments, UK. Crystallographic orientation, in the form of Euler angles (φ1, ɸ, φ2), was recorded for each analyzed point in the investigated samples. Pattern acquisition, band detection, indexing, and recording of the analyzed sample surfaces were automatic. The resulting electron backscattering patterns (EBSPs) or Kikuchi bands were compared with a model crystallographic structure to determine specific crystallographic orientations of the analyzed phases. Diffraction patterns of goethite, hematite, and calcite were determined and data were integrated into crystallographic orientation maps. Data cleaning (removal of wild spikes and zero solution pixels with at least seven neighbors indexed) was done using the AZtecCrystal software. Orientation maps, modal abundance (determined from the area fractions) of the analyzed, indexed phases, and crystallographic orientation analyses were reproduced from the EBSD data (phase information, crystal symmetry, and Euler angles). The low indexing ratio of the EBSD suggests that only the largest goethite grains were analyzed (~13%), indicating a relatively weak LPO. Thresholds were applied with an angle of 10º for grain detection and pole figures were refined to one point per grain for calcite, goethite and hematite.

*XRD* – Samples were crushed first using an agathe mortar and pestel and subsequently pulverized to <10 μm using an agathe ball-milling shaking device. X-ray diffraction analysis of powdered rocks was performed at the University of Lousiana at Lafayette using a *Rigaku* Miniflex 600 benchtop compact diffractometer with D/teX Ultra detector, with a 2θ interval range of 3 to 85º, step width of 0.02º (2θ), a dwell time of 0.15 s/step, maximum voltage of 40 kV, tube current of 15 mA, with a Cu anode (Cu Kα radiation, λ ≈ 1.5406 Å). To identify mineral phases and crystallographic structures, powdered samples were irradiated with X-rays, and the diffracted beams detected to produce diffraction patterns. These patterns were then compared to the International Center for Diffraction Data (ICDD) database for phase identification analysis using the whole powder pattern fitting (WPPF) method in the Rigaku PDXL software (Structure Analysis Wizard).

*Magnetic methods* – The magnetic properties of small rock fragments, measuring 3.5 mm, were determined using a *Princeton Measurements Corporation* Vibrating Sample Magnetometer (VSM) 3900–04 at fields up to 2 T. Magnetic hysteresis curves were corrected for the high-field slope by considering the slope above 70% of the maximum field. The remanent coercivity (Hcr) was determined using backfield experiments involving direct current demagnetization. To conduct a thorough analysis, first-order reversal curve (FORC) analyses were performed using hundreds of curves at field increments of approximately 1.2 mT, up to 120 mT. The FORCInel v.3.08 software^82^ was utilized to process and display the FORC data. Smoothing factors (SF) ranging from 5 to 10 were applied, along with systematic correction of the first point artifact. Small cubes (~40 mm^3^), prepared from the AMS study, underwent stepwise, room temperature, isothermal remanent magnetization (IRM) using the VSM, up to 2.0 T. The magnetic components, including mineral phase and percentage, were evaluated using the cumulative log-Gaussian analysis provided by the MAX Unmix software^83^.

*AMS -* AMS parameters and directions were determined on 3.5 mm cubes at room temperature (~20±1ºC ) using a Kappabridge KLY-4S susceptometer in spinning mode with the SUFAR Agico software^94^ and the protocol of ^85^. The use of the AMS method on smaller cubes (3.5 mm) than in regular studies (20 mm) is justified by the material’s fine grain size, which renders the fabric representative, and by signal stacking using a larger number of samples^86^. The applied field was 450 A/m to increase the signal to noise ratio, with an alternating field at 875 Hz frequency. Directional reproducibility of the results was evaluated through 10 successive measurements on a single specimen. The AMS directions of K_1_, K_2_, and K_3_ proved to be reproducible within 2º. K_1_ is referred to as magnetic lineation and K_3_ is the pole to the magnetic foliation. We used the Anisoft 5.1.03^87^ software to display and process the results. The parameters calculated from the data include mean magnetic susceptibility (arithmetic mean of the three principal axes, *K_1_*, *K_2_* and *K_3_*, defined in^88^)

*K_m_ = (K_1_ + K_2_ + K_3_) / 3*  Equation S1

the corrected degree of anisotropy^88^

*P’=*$exp\sqrt{\{2[\left( n_{1}-n_{m} \right)^{2}+\left( n_{2}-n_{m} \right)^{2}+\left( n_{3}-n_{m} \right)^{2}]\}}$ Equation S2

and the shape parameter^88^

$T=\left[ \frac{2(n_{2}-n_{3})}{{(n}_{1}-n_{3})} \right]-1$ Equation S3

where *n* is the natural logarithm of the anisotropy principal axes (*e.g.*, *n*_1_= ln *K_1_*),

and $n_{m}=\sqrt[3]{n_{1}\cdot n_{2}\cdot n_{3}}$.

*Thermomagnetic experiments -* The variations of low-field magnetic susceptibility as a function of temperature and Curie temperature (T_c_) were investigated in the 25-700ºC range in argon flux, using a CS-4 furnace coupled to a Kappabridge KLY-4 instrument moderately crushed samples of the AMS cubes. We used the first derivative of the curve to determine T_c_^95^. Additional thermomagnetic/baking experiments were performed through flash heating in periods of 120 seconds in steps of 25ºC up, followed by cooling to room temperature, up to 700ºC. The specimens were powdered, encased in a fused silica vial, and baked in the air at ambient pressure to test the stability of mineral phases, while magnetic susceptibility was measured after each step.

Rock Magnetism results

The respective contributions of minerals to magnetic susceptibility are calculated using^89^ method, mineral contents from X-ray diffraction and published values for intrinsic volume-normalized magnetic susceptibility, κ (Table S1). The magnetic susceptibility is dominated by the two antiferromagnetic phases: goethite and hematite. The proportions of diamagnetic minerals (calcite, quartz, dolomite, and kaolinite) and antiferromagnetic phases (goethite, hematite) in the three slabs of the hematite fault mirror (Figure 1D) are given in Table S1. Table S1 also provides each mineral's contribution to magnetic susceptibility, calculated using XRD modal percentages and intrinsic magnetic properties. These calculations highlight goethite's dominant contribution (65 to 100%) to magnetic susceptibility across all slabs and hematite's significant role (~40%) in the fault mirror slab. Furthermore, FORC analysis (Figures 3C, D, E) indicates that goethite and hematite occur in the superparamagnetic (SP) domain state, corresponding to physical sizes ~30 nm for hematite^34^ and ~25 nm for goethite^49,50^. High-field magnetic hysteresis results confirm that superparamagnetic phases (goethite and hematite together) comprise ~66% of the low-field magnetic susceptibility in the hematite-rich fault mirror. Finally, IRM acquisition data processed with the MaxUnmix IRM deconvolution software^83^ estimate the contributions of goethite (B_h_ ~870 mT) and hematite (B_h_ ~104 mT) to magnetic remanence at ~ 91% and ~9%, respectively (Supp. Mat., Figure S4).

Anisotropy of Magnetic Susceptibility (AMS)

We measured the AMS of 115 oriented 3.5 mm cubes cut from three 3.5 mm-thick juxtaposed slabs parallel to the fault mirror (Figure 2F) and report the Jelinek (1981) parameters below: i) hematite-rich fault mirror (h in Figure 2F), ii) goethite-rich slab 2 (g in Figure 2F), and iii) goethite-rich slab 3 (g in Figure 2F). Overall, the mean magnetic susceptibility (K_m_) ranges widely from 3 to 321 x 10^-6^ [SI] with the following means and standard deviations: i) hematite-rich fault mirror, K_m_ = 163 ± 45 x 10^-6^ [SI], ii) goethite-rich slab 2, K_m_ = 156 ± 38 x 10^-6^ [SI], and iii) goethite-rich slab 3, K_m_ = 73 ± 56 x 10^-6^ [SI]. The total contribution of diamagnetic minerals to AMS (K_DIA ~_ -11.6 x 10^-6^ [SI]) was corrected for, as shown in Supp. Mat. - Table S1, to calculate the corrected degree of magnetic anisotropy as P’ = (K_max_ - K_DIA_) / ( K_min_ - K_DIA_) (Supp. Mat. – Table S2). Overall, P’ ranges from 1.106 to 1.491 with a median of 1.204 ± 0.085. These relatively high P’ values indicate that ferromagnetic phases dominate the AMS^90^. The three slabs show the following means and standard deviations: i) hematite-rich fault mirror, P’ = 1.137 ± 0.053, ii) goethite-rich slab 2, P’ = 1.242 ± 0.073, and iii) goethite-rich slab 3, P’ = 1.242 ± 0.079. The two goethite-rich slabs have a stronger anisotropy than the hematite-rich slab, due to the higher degree of anisotropy of goethite. The magnetic shape parameter T ranges from -0.484 to 0.530 with a median of 0.171 ± 0.254 and the following values for the three slabs: i) hematite-rich fault mirror, T = 0.236 ± 0.308, ii) goethite-rich slab 2, T = 0.128 ± 0.216, and iii) goethite-rich slab 3, T = 0.142 ± 0.231.

*AMS fabrics and directions* – The low-field principal AMS directions are bootstrapped and plotted in geographic framework stereonets (Figures 3F, G, and H). Both bootstrapped and raw directional data (Figure Suppl. Mat. S5) are spatially homogeneous and show magnetic fabric consistency at the scale of a few centimeters. For the fault mirror (Figure 3F) in which AMS is controlled by SP hematite, K_3_ axes, poles to magnetic foliation, plot close to the normal to the fault plane (N120º, 66º SW) and K_1_ axes, magnetic lineation (N198º, 58º) plot close to the slickenlines direction (N210º, 66º).

Rock-Eval results

*Rock-Eval*, a well-established pyrolysis method, widely used in oil and gas exploration, consists of stepwise combustion of organic matter through heating a rock specimen in an anoxic environment^35,36^. This pyrolysis takes place within a minute, thus at timescales comparable to the duration of seismic slip. We apply this method to test for a possible thermal anomaly across the carbonate fault mirror. We selected an area away from goethite deposits where hydrocarbons might have been transported by fluids. The results, based on ten samples collected from two 10 mm diameter cores 10 mm away from each other, document a substantial decrease in hydrocarbon contents (S1, S2, and S3) by 55 to 30% between 1.5 and 7.5 mm away from the fault mirror (Supplemental Material - Figure S7). This decrease cannot be explained by either i) pre-existing compositional differences in the limestone because the analyzed carbonate is chemically homogeneous, as shown by its inorganic carbon content, or by ii) leaching by iron-rich fluids although this scenario is unlikely since organic matter, which is insoluble in water, would not easily be transported. The most likely explanation for the observed RockEval anomaly, close to the HLF mirror, is that hydrocarbons were pyrolyzed into CH_4_ due to localized frictional heating (reaching 120ºC, at 4.5 mm from the slip plane).

Figure S1A.

Geological cross-section of the Hebgen Lake Fault area showing the location of Laramide-age thrusts (black) and Hebgen Lake normal fault (red) (after^23^).


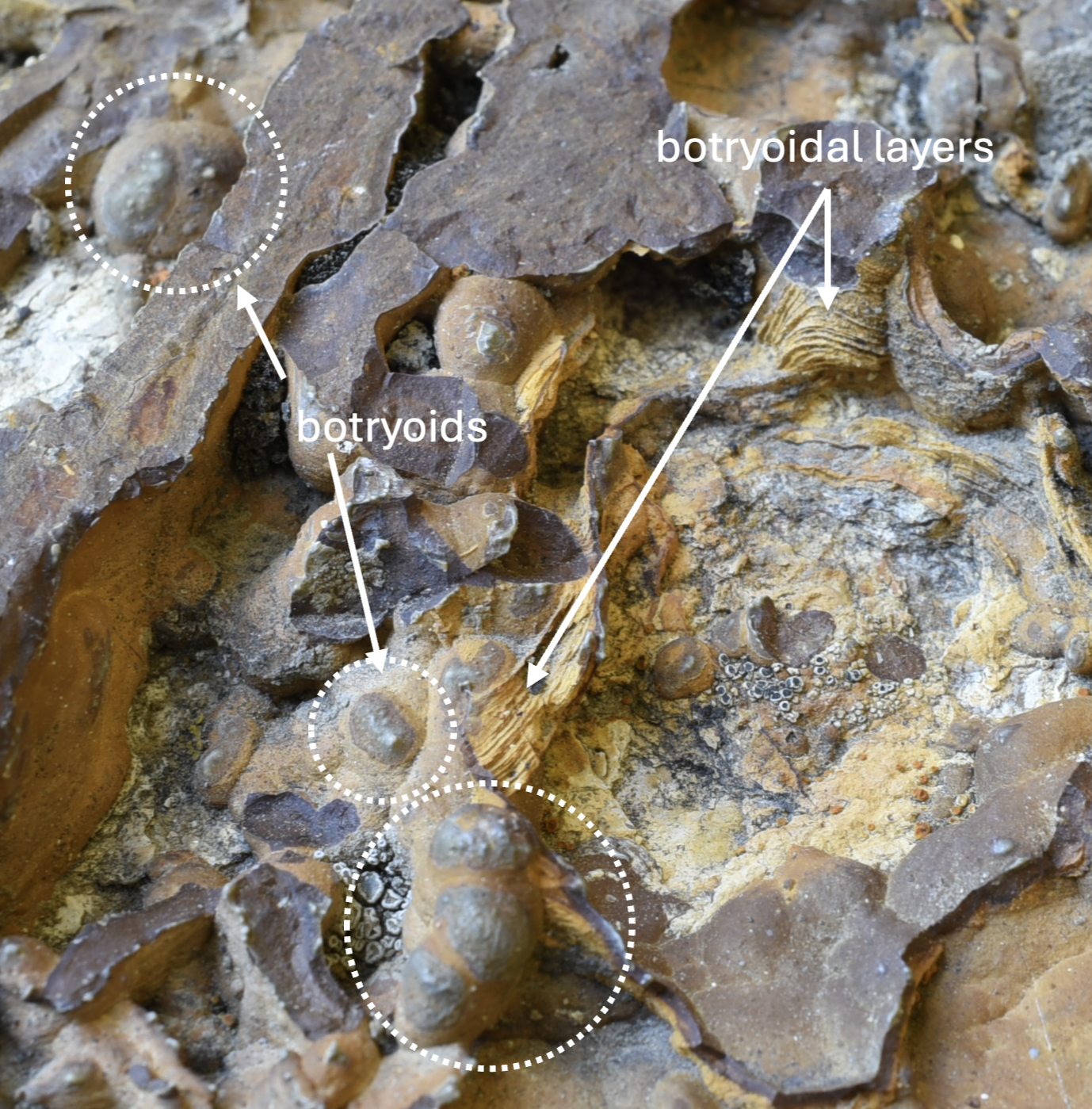


Figure S1B.

Botryoidal layers cut by erosion showing their depositional internal structure and botryoids showing their bulbous macroscopic morphology at the S31 Creek bedrock fault scarp. The botryoidal layered macrostructures could not have formed during fault seismic slip or creep of the fault and therefore must have formed during aseismic / interseismic periods


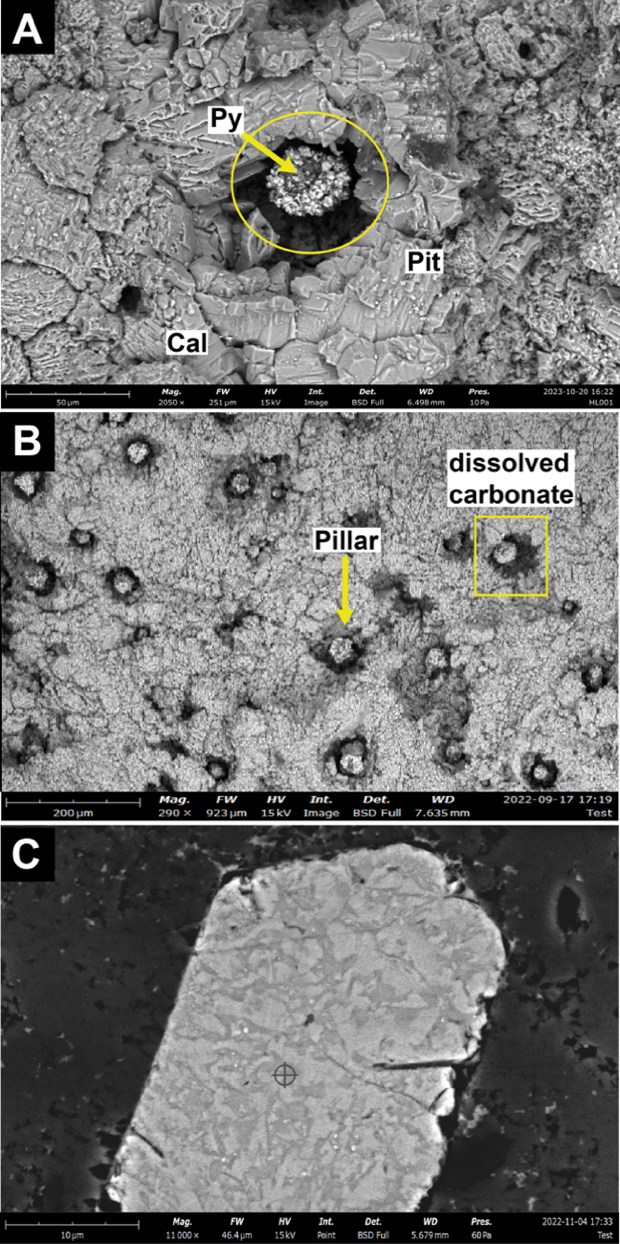


Figure S2.

Formation mechanism of morphological pits in carbonates on A1 mirror.

A. Pyrite grain in carbonate matrix showing a topographic depression around the sulfide, interpreted as result of carbonate dissolution by sulfuric acid with the reactions:

4 FeS_2_ + 11 O_2_ → 2 Fe_2_O_3_ + 8 SO_2_ *Equation S4*

2 SO_2_ + 2 H_2_O + O_2_ → 2 H_2_SO_4_ *Equation S5*

H_2_SO_4_ + CaCO_3_ → CaSO_4_ + H_2_O + CO_2_ *Equation S6*

Fe_2_O_3_ + H_2_O → 2 FeO(OH) *Equation S7*

159.69 g + 18.01 g → 177.7 g

**B**. Multiple morphological pits in carbonate attest that sulfide leaching locally dissolves carbonate, liberates iron, and produces hematite and goethite. Detail of a morphological pit showing central pillars of Fe-Mn-rich carbonate.

**C**. Near the fault mirror (<75 μm), a pyritohedron pyrite grain show heterogeneous internal microstructures indicative of incipient thermal destabilization. These microstructures are not found elsewhere.

**
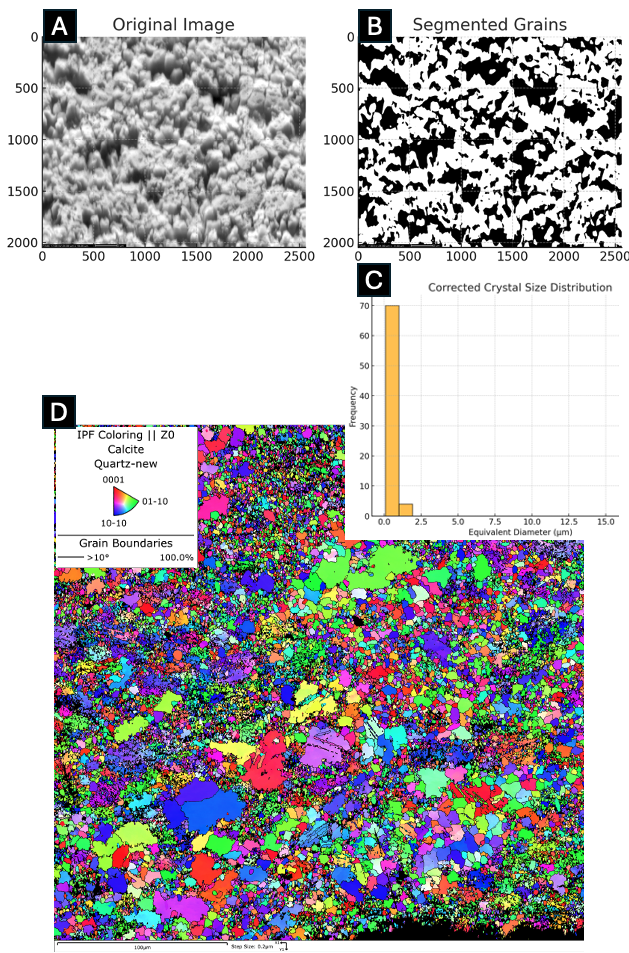
**

Figure S3.

Figure S3. (A). Scanning electron microscopy image (topography) showing nanocalcite layer grain morphology in A1 carbonate fault mirror; (B) Segmented grains from crystal size distribution using Fiji software^96^; (C) Crystal size distribution showing uniform grain size and slight asymmetry towards larger sizes; (D) Electron BackScattered Diffraction indexed pole figures in A3 hematite fault mirror. The fault mirror is at the bottom of the image.


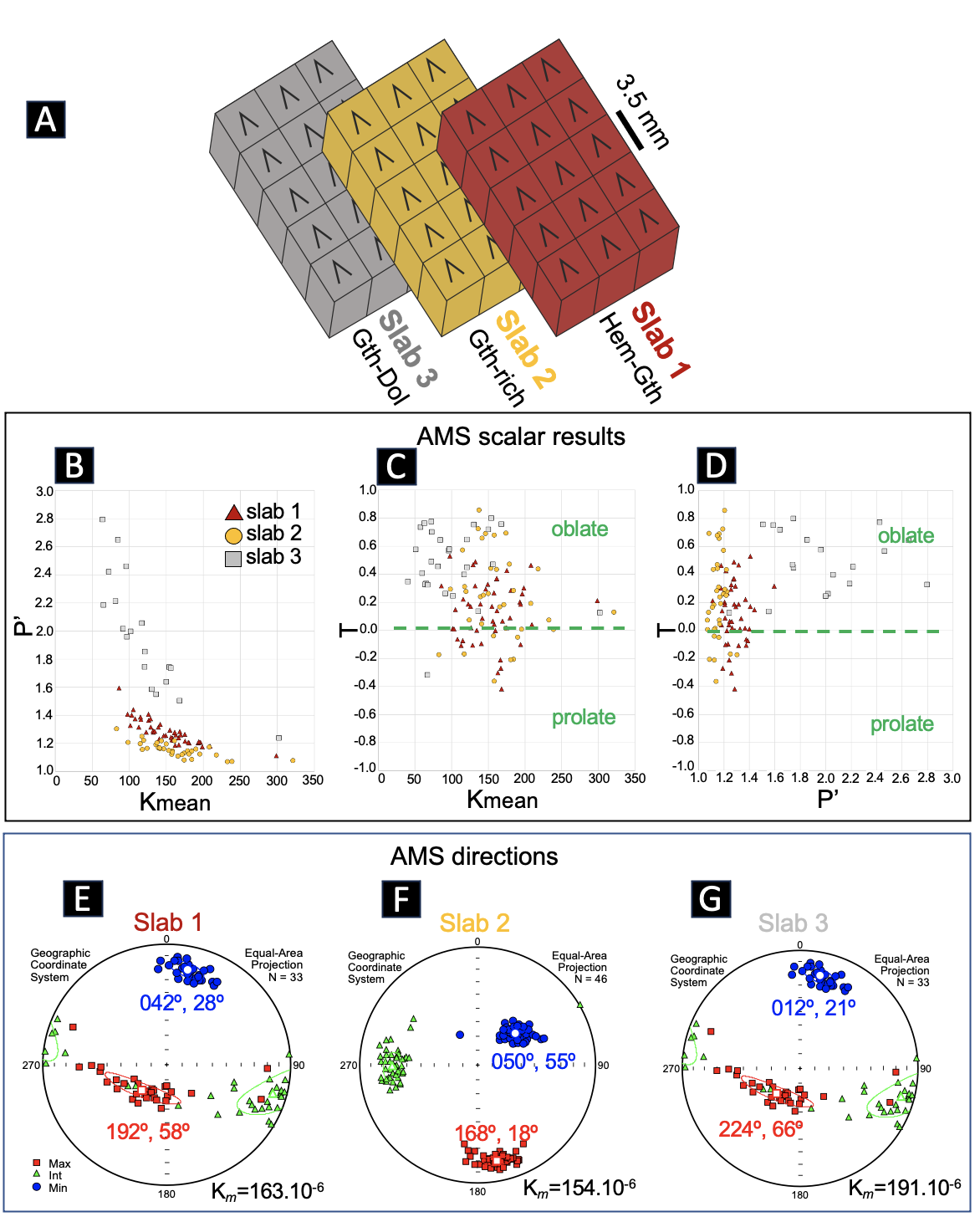


Figure S4.

**A.** Spatial organization of the mini AMS samples with respect to hematite (A3) fault mirror. **B, C,** and **D.** AMS scalar parameters showing the mean magnetic susceptibility (K_mean_), the degree of anisotropy (P’), the shape parameter (T), and stereonets of directional data for all three layers of hematite fault mirror showing the consistency of AMS direction with slip plane and down-dip direction.

**
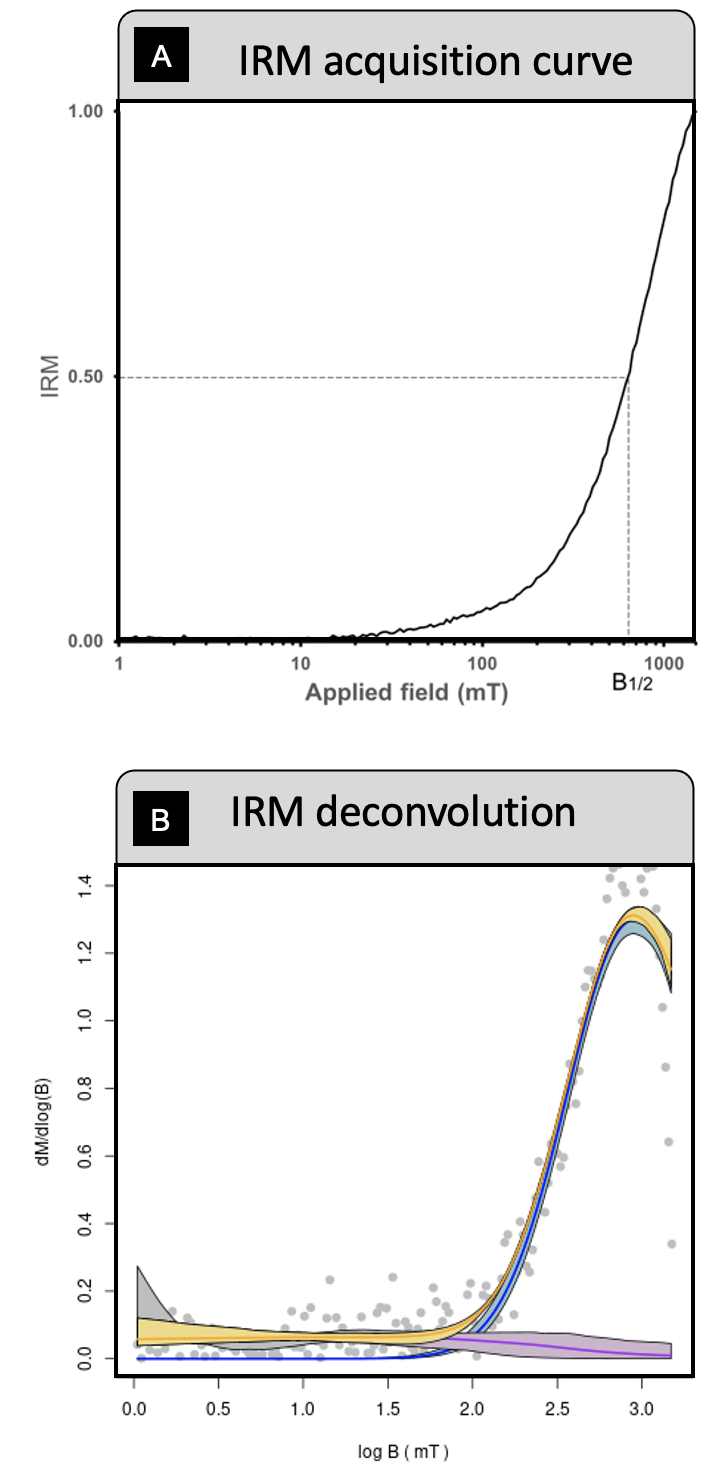
**

**Figure S5.**

Normalized isothermal remanent acquisition (IRM) experiment. B. IRM deconvolution using MaxUnmix^83^. The IRM is dominated by one magnetically remanent phase.

**
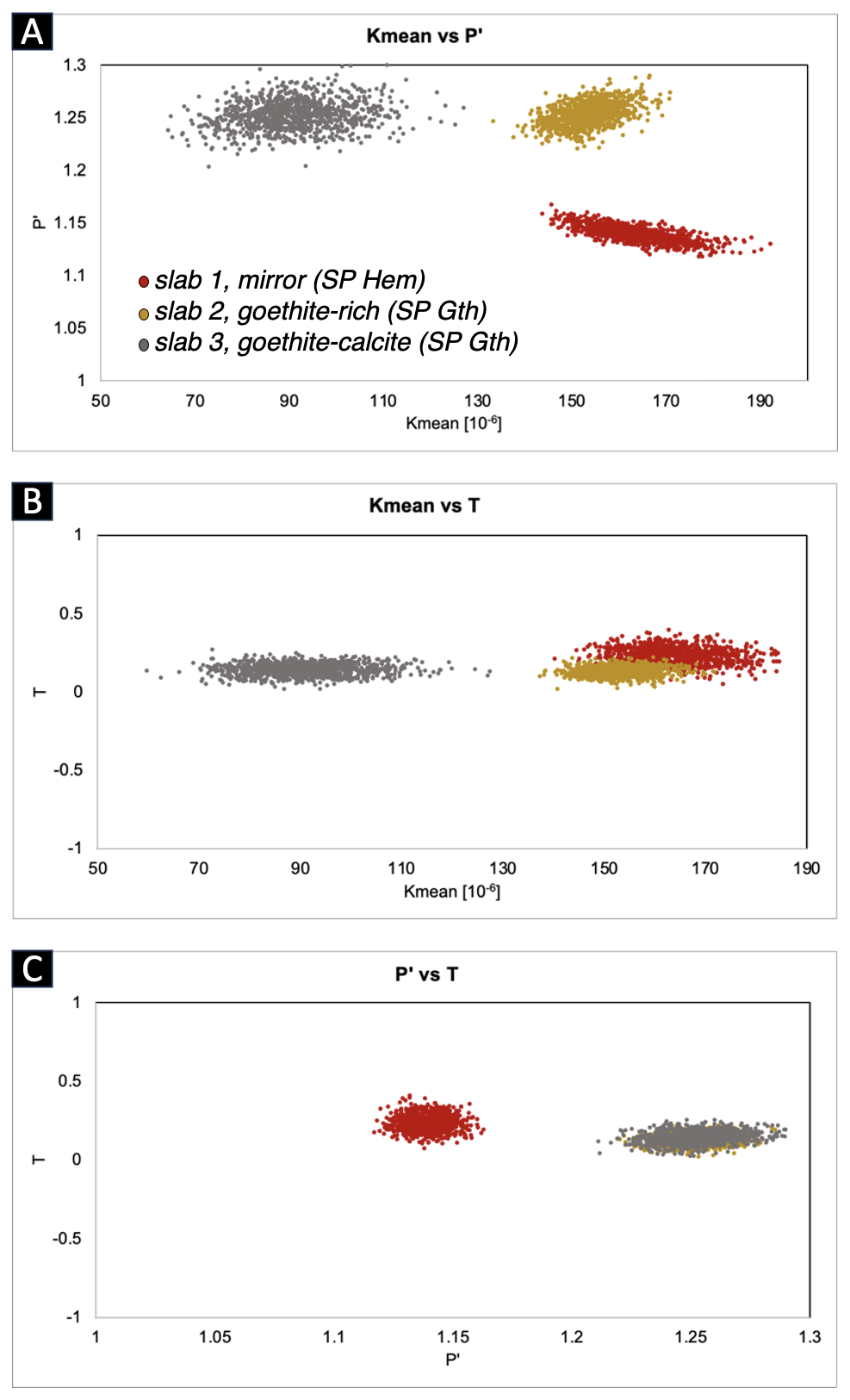
**

**Figure S6.**

Bootstrapped AMS data for the three layers of the HLF hematite fault mirror.

**
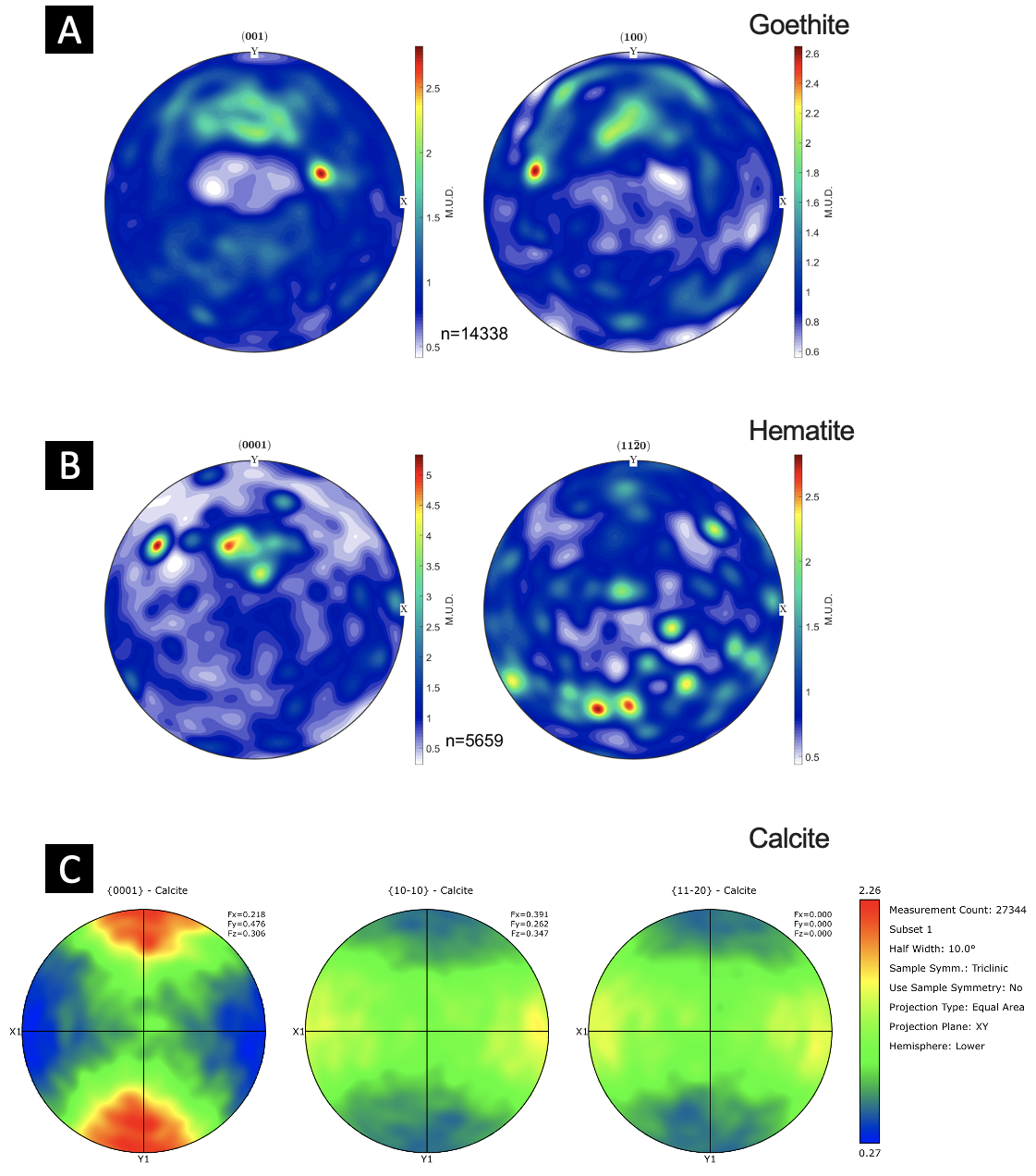
**

**Figure S7.**

Electron backscattered diffraction data for main crystallographic axes of goethite, hematite and calcite in A3 hematite fault mirror (layer 1). A. Maximum point distribution of goethite (001) and (100) crystallographic axes. B. Crystallographic axes (0001) and (11‾20) form partial girdle distribution indicative of partial activation of multiple slip systems. C. Point maxima distribution of calcite (0001) axes indicative of low strain, possibly indicative of recrystallization under thermal gradient. Lack of CPO in other crystallographic directions rules out synkinematic recrystallization.

**
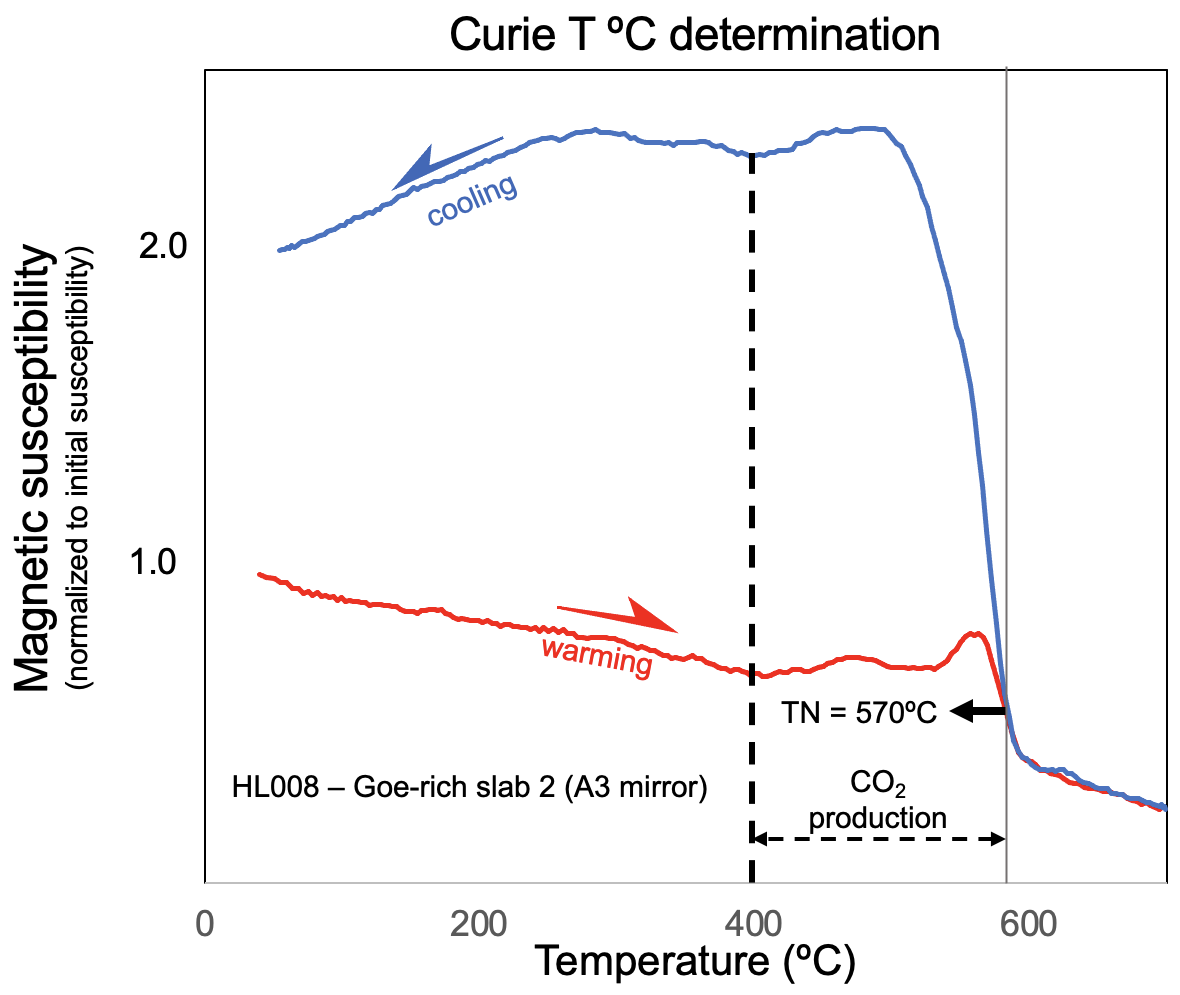
**

**Figure S8.**

Thermomagnetic experiment on goethite-rich slab 2 of a hematite mirror (A3) showing the high Curie temperature (570ºC) related to low-Ti hematite. The moderate decrease in magnetic susceptibility up to 400ºC is due to goethite dehydration. Above ~400ºC, goethite reacts to hematite as shown by the decrease in magnetic susceptibility.

**
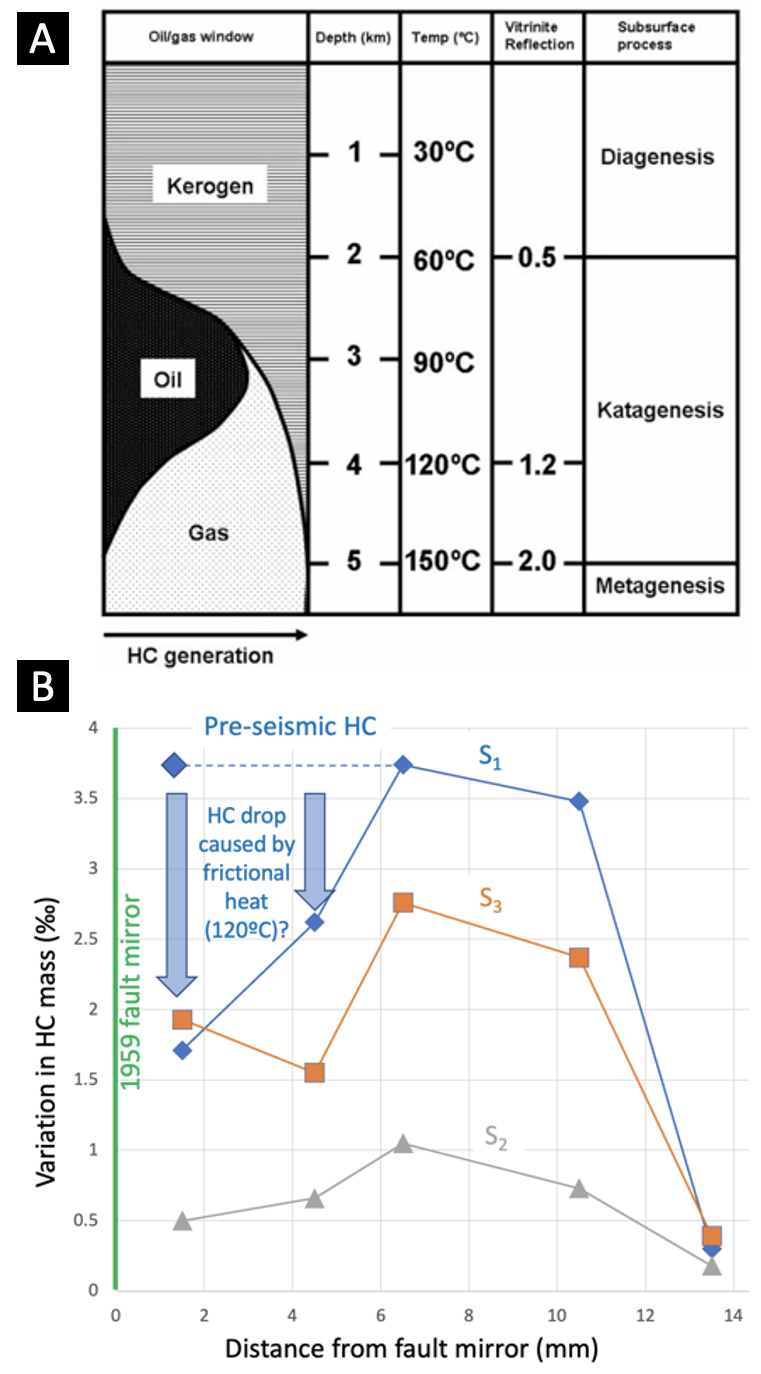
**

**Figure S9.**

A. General fields of organic matter stability based on the Rock-Eval method^35^. B. Rock-Eval experiment for Hebgen Lake Fault carbonate mirror A1 showing loss of hydrocarbon (HC) due to frictional heat recorded at 4.5 mm distance from the slip surface. The main Rock-Eval plotted parameters are S_1_ (HC amount already present in the sample), S_2_ (HC yield formed by thermal cracking of kerogen), and S_3_ (CO_2_ yield formed during pyrolysis).


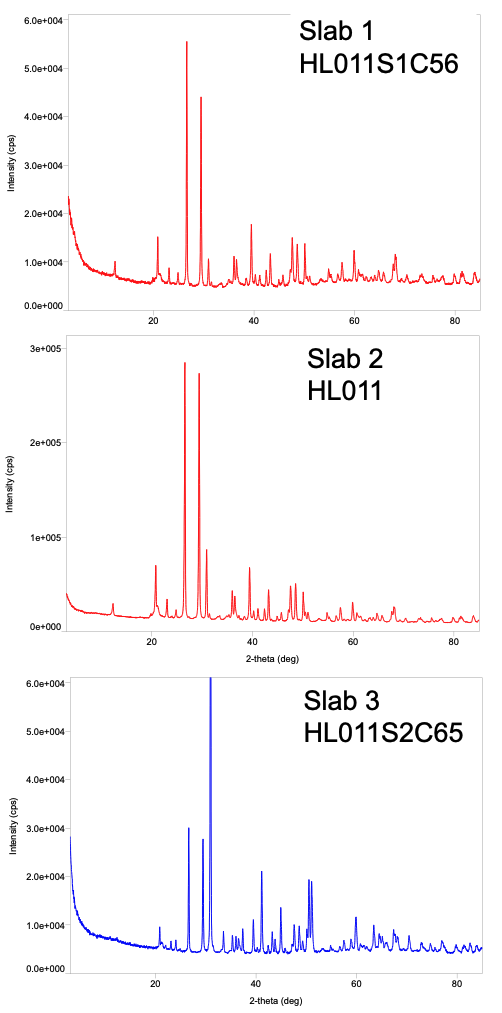


**Figure S10**. X-ray diffraction patterns for hematite fault mirror A3 (top: mirror, middle: slab 2, bottom: slab 3) corresponding to the data of Table S1.


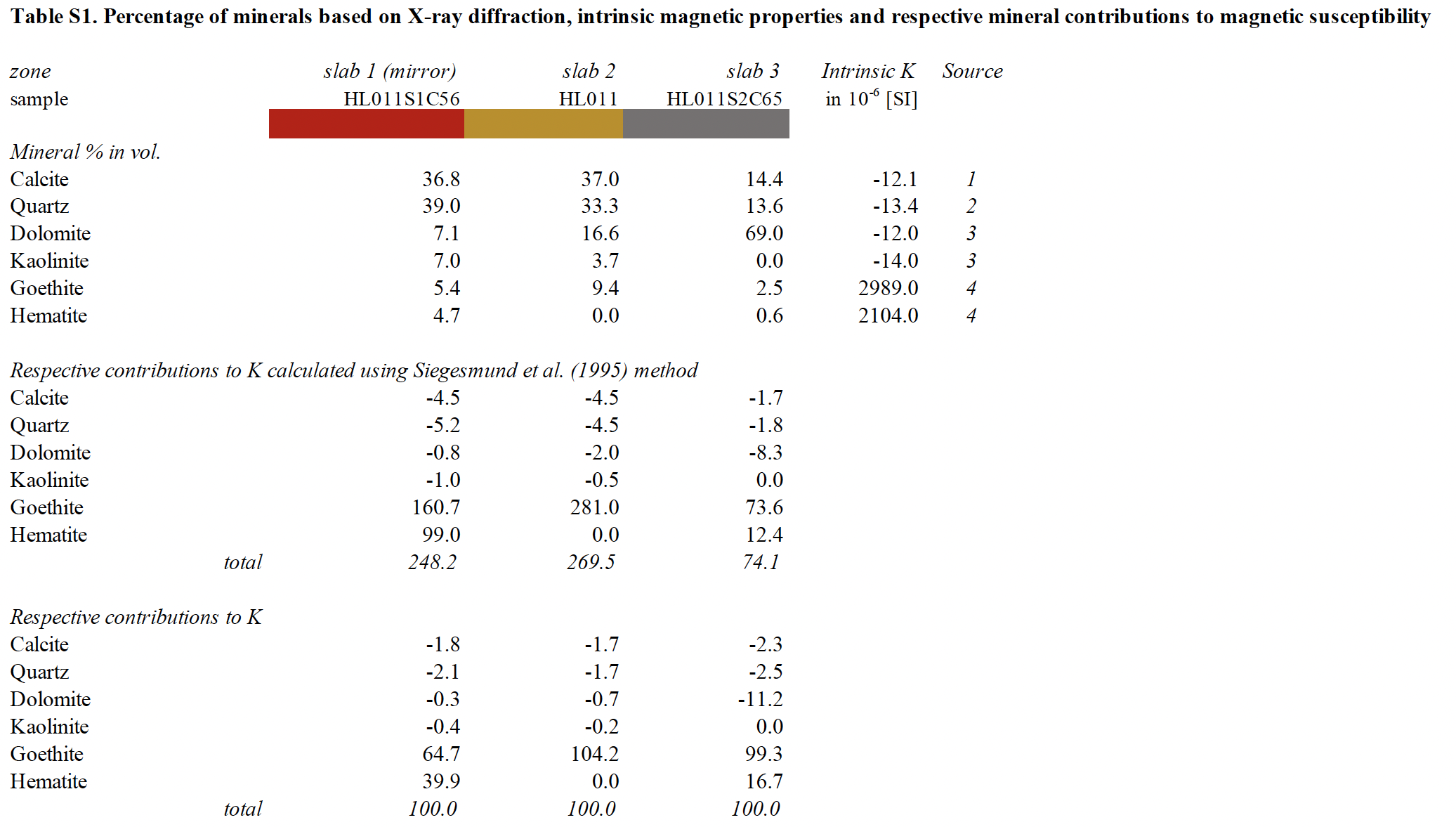


**Table S1.**

Percentage of minerals determined from X-ray diffraction, intrinsic magnetic properties and respective mineral contributions to magnetic susceptibility.

**Supplemental Materials References**

(82) Harrison, R.J. & Feinberg, J.M. FORCinel: an improved algorithm for calculating first‐order reversal curve distributions using locally weighted regression smoothing. Geochem. Geophys. 9, doi:10.1029/2008GC001987 (2008).

(83) Maxbauer, D.P., Feinberg, J.M. & Fox, D.L. MAX UnMix: a web application for unmixing magnetic coercivity distributions. Comput. Geosci. 95, 140–145 (2006).

(84) Jelínek, V. & Pokorný, J. Some new concepts in technology of transformer bridges for measuring susceptibility anisotropy of rocks. Phys. Chem. Earth 22, 179–181 (1997).

(85) Ferré, E.C., Chou, Y.M., Kuo, R.L., Yeh, E.C., Leibovitz, N.R., Meado, A.L., Campbell, L. & Geissman, J.W. Deciphering viscous flow of frictional melts with the mini-AMS method. J. Struct. Geol. 90, doi:10.1016/j.jsg.2016.07.002 (2016).

(86) Zamanialavijeh, N., Hosseinzadehsabeti, E., Ferré, E.C., Hacker, D.B., Biek, R.F. & Biedermann, A.R. Kinematics of frictional melts at the base of the world’s largest terrestrial landslide: Markagunt Plateau, southwest Utah, United States. J. Struct. Geol. 153, doi:10.1016/j.jsg.2021.104448 (2021).

(87) Chadima, M. Anisoft Advanced Treatment of Magnetic Anisotropy Data. MS Windows software. Version 5.1.03 (2019)

(88) Jelinek, V. Characterization of the magmatic fabrics of rocks. Tectonophysics 79, 63–67 (1981).

(89) Siegesmund, S., Ullemeyer, K. & Dahms, M. Control of magnetic rock fabrics by mica preferred orientation: a quantitative approach. J. Struct. Geol. 17, 1601–1613 (1995).

(90) Rochette, P., Jackson, M. & Aubourg, C. Rock magnetism and the interpretation of anisotropy of magnetic susceptibility. Rev. Geophys. 30, 209–226 (1992).

(91) Schmidt, V., Günther, D. & Hirt, A.M. Magnetic anisotropy of calcite at room temperature. Tectonophysics 418, 63–73 (2006).

(92) Tarling, D. & Hrouda, F. The magnetic anisotropy of rocks. Chapman, London, 217 p. (1993).

(93) Maher, B.A. Magnetic properties of modern soils and Quaternary loessic paleosols: paleoclimatic implications. Palaeogeogr. Palaeoclimatol. Palaeoecol. 137, 25–54 (1998).

(94) Jelínek, V. and Pokorný, J., 1997. Some new concepts in technology of transformer bridges for measuring susceptibility anisotropy of rocks. *Physics and Chemistry of the Earth*, *22*(1-2), pp.179-181.

(95) Fabian, K., Shcherbakov, V.P. and McEnroe, S.A., 2013. Measuring the Curie temperature. *Geochemistry, Geophysics, Geosystems*, *14*(4), pp.947-961.

(96) Schindelin, J., Arganda-Carreras, I., Frise, E., Kaynig, V., Longair, M., Pietzsch, T., Preibisch, S., Rueden, C., Saalfeld, S., Schmid, B. and Tinevez, J.Y., 2012. Fiji: an open-source platform for biological-image analysis. *Nature methods*, *9*(7), pp.676-682.
